# Molecular Atlas of Key Food Odorants Reveals Mixture‐Level Organization and Enables Generative Aroma Design

**DOI:** 10.1002/advs.77740

**Published:** 2026-09-13

**Authors:** Jingzhi Zhang, Huadong Xing, Antonella Di Pizio, Qinfei Ke, Xingran Kou, Dachuan Zhang

**Affiliations:** ^1^ Collaborative Innovation Center of Fragrance Flavour and Cosmetics Faculty of Flavour Fragrance and Cosmetics Shanghai Institute of Technology Shanghai China; ^2^ Department of Food Science and Technology Faculty of Science National University of Singapore Singapore Singapore; ^3^ Empa−Swiss Federal Laboratories for Materials Science and Technology Technology and Society Laboratory Gallen Switzerland; ^4^ Leibniz Institute for Food Systems Biology at the Technical University of Munich Freising Germany; ^5^ Chemoinformatics and Protein Modelling TUM School of Life Sciences Technical University of Munich Freising Germany; ^6^ Bezos Centre for Sustainable Protein at the National University of Singapore Singapore Singapore; ^7^ National University of Singapore (Suzhou) Research Institute Jiangsu China

**Keywords:** food flavor, machine learning, odor perception, sensory science, systems biology

## Abstract

Aromas arise from complex combinations of odorants, yet how these mixtures encode stable and recognizable aroma identity remains unclear. Resolving this gap is key to both basic olfaction research and translational aroma design. Food provides a unique real‐world system in which diverse mixtures produce well‐defined aroma identities. Here we present KFO‐Atlas, a molecular atlas of 896 key odorants curated from 2,282 food‐derived aroma profiles. Analysis shows that every measured food aroma comprises at least three key odorants. Plant‐derived food mixtures generally exhibit greater diversity in key odorant composition than animal‐derived foods. Notably, in certain cases, distinct systems (e.g., plant‐ and animal‐based foods) converge on a similar key odorant composition via shared reaction pathways. Building on these insights, we develop KFO‐Gen, a generative AI model that produces category‐targeted aroma formulations and validate its outputs by blinded human sensory evaluation. As a proof of principle, the model reconstructs meat‐like aromas using exclusively plant‐derived odorants, highlighting the potential of AI‐guided aroma design for sustainable food innovation. KFO‐Atlas and KFO‐Gen establish a foundation for mixture‐level studies of aroma, advancing both fundamental understanding and generative design.

## Graphical abstract

KFO‐Atlas reveals how real‐world aromas are organized as structured mixtures rather than individual molecules. Building on these principles, KFO‐Gen, a generative AI framework, designs perceptually valid aroma formulations and reconstructs meat‐like aromas exclusively from plant‐derived odorants, providing a foundation for mixture‐level studies and AI‐guided aroma design.

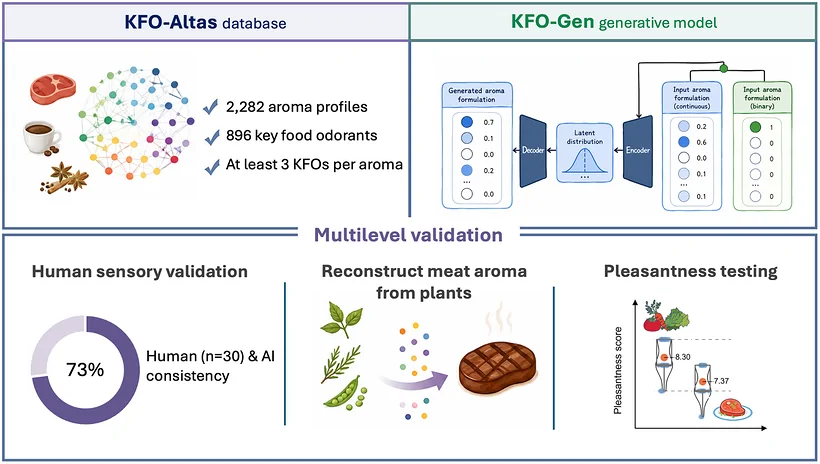

## 1 Introduction

Olfaction translates chemical signals into perception and behavior, shaping choice, memory, and affect [1, 2]. While progress has been made in linking molecular features to the perception of individual odorants, most natural olfactory stimuli are complex mixtures rather than single molecules [3]. How the composition of such mixtures gives rise to stable and recognizable perceptual identities—here referred to as aromas—remains an unresolved problem.

Food‐derived odors provide a uniquely rich and experimentally grounded model system to study this problem. Across cultures and contexts, foods comprise diverse yet reproducible odorant mixtures that give rise to stable aroma identities, such as chocolate or wine [4]. Importantly, these mixtures span a wide range of chemical complexity while remaining perceptually meaningful, making food a real‐world testbed for investigating how mixture composition shapes aroma identities [5].

Although over 10,000 volatile compounds have been identified in foods, olfactory perception is not determined by the full volatile repertoire but is dominated by a much smaller subset of odor‐active molecules [5, 6]. These key food odorants (KFOs), defined as volatile compounds with odor activity values (OAVs) greater than one, exert a disproportionate influence on perceived aroma identities [6, 7]. As such, KFOs provide a natural molecular abstraction of complex odorant mixtures, capturing the components most relevant to aroma organization while reducing chemical dimensionality.

Early compilations estimated that only a small fraction of volatiles—approximately 230 compounds (less than 3%)—qualify as KFOs [7], although this number likely underrepresents true odorant diversity given substantial advances in analytical sensitivity over the past decade. Meanwhile, as studies of odorant mixtures have expanded across diverse matrices and processing conditions, KFO data have accumulated in a fragmented manner [8]. Consequently, the field lacks an integrated landscape of odor‐active compounds that would enable systematic comparisons across real‐world mixtures and support a statistical understanding of how aroma organization emerges.

This gap also constrains aroma design. Despite the importance of KFO being well recognized, aroma perception arises from nonlinear interactions among multiple odorants [9], resulting in a vast combinatorial space that cannot be exhaustively explored experimentally [10]. Existing computational approaches have largely focused on predicting sensory attributes of individual odorants [11, 12, 13] or have been restricted to a small number of well‐studied product categories, such as wine or whisky [14, 15]. As a result, a general framework that links empirically observed odorant co‐occurrence patterns to controllable aroma formulation remains lacking, constraining both fundamental efforts to uncover mixture organization and applied efforts toward rational aroma design.

Here, we introduce a conceptual and methodological framework to decode the molecular organization of real‐world odorant mixtures using food aroma as a model system. We construct KFO‐Atlas, a comprehensive database of 2,282 food‐derived aroma profiles. Using this resource, we systematically examine how odorant combinations are organized across mixtures, and whether common structural patterns underpin aroma identity and similarity. In particular, we ask whether the frequency with which individual odorants appear across mixtures reflects their functional roles in shaping perceptual identity. We further explore the extent to which distinct mixture systems (such as plant‐ and animal‐based foods) may converge on similar KFO composition under shared reaction pathways. Building on this organization, we develop KFO‐Gen based on a conditional variational autoencoder to test whether structured knowledge of odorant combinations can be translated into controllable aroma formulation. We validate the perceptual category fidelity of the generated formulations through a blinded sensory evaluation, in which 30 experienced panelists assessed 100 AI‐generated formulations for their perceived aroma category. As a proof of principle, we further apply the framework to generate meat‐like aromas using exclusively plant‐derived odorants.

## 2 Results

### 2.1 Every Measured Food Aroma Comprises at Least Three Key Odorants

**FIGURE 1:**
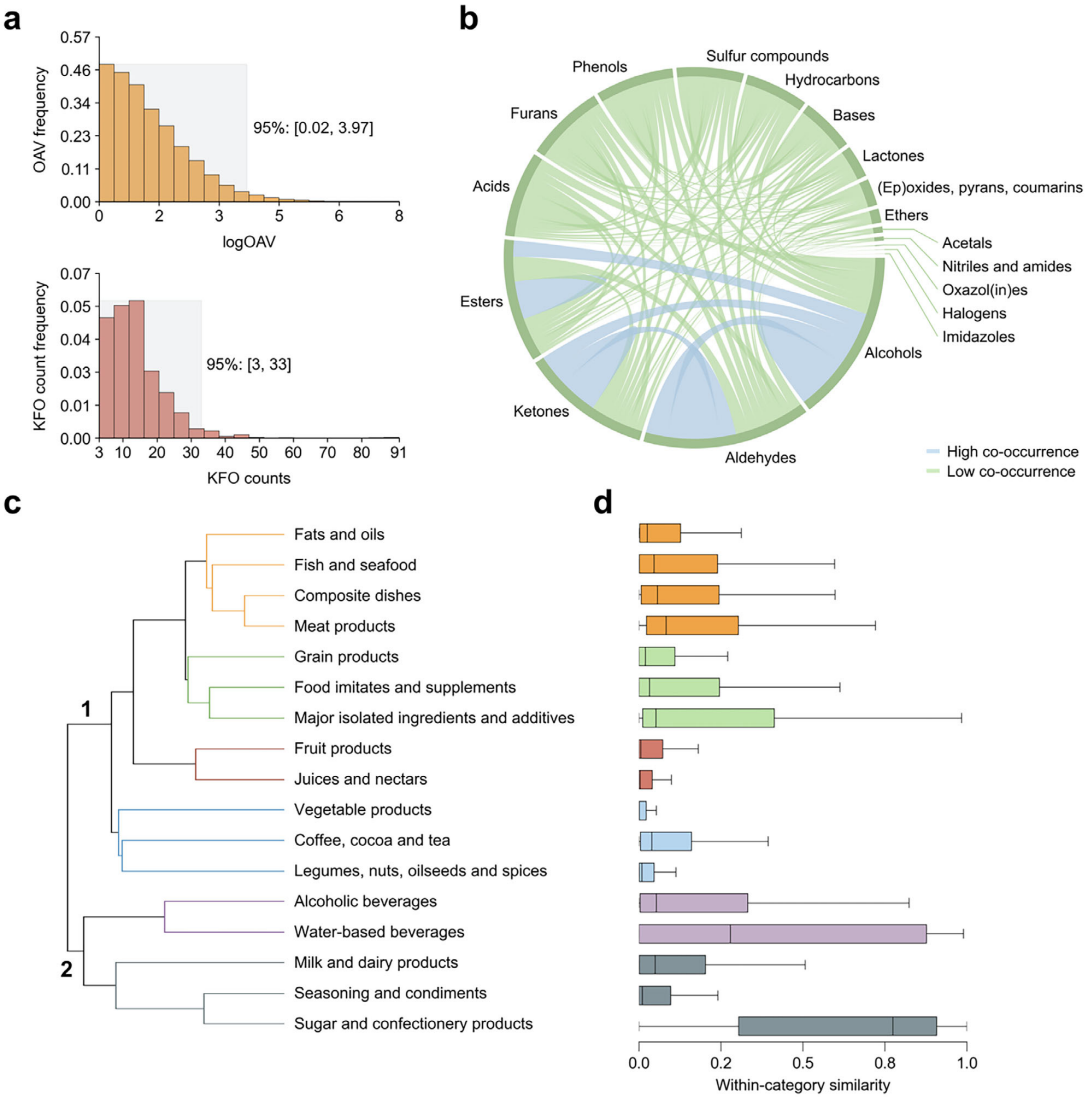
Sparse combinations of key food odorants (KFOs) underlie real‐world food aromas. Aromas are built from sparse combinations of KFOs that give rise to structured organization across chemical classes and aroma categories. (a) Distribution of the number of KFOs per aroma profile and the corresponding odor activity values (OAVs) of individual KFOs. Notably, all aroma profiles in KFO‐Atlas include at least three KFOs. The blue contour encloses the central 95% of observations. (b) Network representation of chemical co‐occurrence across aroma profiles, in which nodes represent KFO chemical groups and edges denote co‐occurrence frequency. The width of the outer arcs reflects the total co‐occurrence of each chemical group across profiles. Edge colors indicate co‐occurrence frequency, categorized using a threshold of 1,000 joint occurrences to distinguish high‐ and low‐frequency co‐occurrences. (c) Hierarchical clustering of 17 major food categories with at least ten profiles based on the cross‐category similarity of their KFO compositions, showing that aromas organize into distinct groups in KFO composition space. (d) Distributions of within‐category pairwise similarity scores among KFO profiles for each category. Lower similarity scores indicate greater KFO compositional heterogeneity, whereas higher scores reflect stronger convergence toward shared KFO compositions. Boxplots display the median and interquartile range, with whiskers extending to 1.5× the interquartile range.

KFO‐Atlas compiles 2,282 food‐derived aroma profiles curated from publications (2015–2024), covering 20 food categories and 86 subcategories based on FoodEx2 [16] classification (Data S1). Across all profiles, we identified 896 unique KFOs, with the central 95% of OAVs spanning 1.05 to 9,332.54. Despite this broad chemical repertoire, individual profiles relied on only a limited subset of KFOs: the central 95% of profiles contained between 3 and 33 odorants (Figure 1a). Notably, no aroma profile in KFO‐Atlas was characterized by fewer than three KFOs. This indicates that real‐world aroma is a combinatorial property rather than a single‐molecule trait.

KFOs were classified into 18 chemical groups based on molecular structure (see *Methods*). Esters (20%), alcohols (13%), aldehydes (11%), ketones (11%), sulfur compounds (10%), and hydrocarbons (9%) accounted for most identified KFOs (Table S1). However, chemical abundance alone did not fully explain their roles in aroma organization. In the co‐occurrence network, alcohols and aldehydes emerged as the most connected nodes, indicating they may act as connectors between diverse aroma compositions (Figure 1b). Consistent with this, most co‐occurrence relationships (71%) occurred between different chemical classes rather than within the same class, suggesting that real‐world aromas are built from mixtures of chemically distinct odorants rather than from single‐class combinations.

The number of KFOs per aroma profile varied across food categories (Table S2). Categories such as coffee, cocoa, and tea (mean 16.49), seasonings and condiments (17.05), and legumes, nuts, oilseeds, and spices (17.02) showed the highest KFO counts, whereas water‐based beverages (9.36), fish and seafood (10.63), and dairy products (11.29) exhibited much lower numbers of KFOs. Collectively, these analyses establish a quantitative baseline for comparing aroma composition and for linking sparse KFO sets to aroma identity.

### 2.2 Plant‐ and Animal‐Derived Foods Exhibit Distinct Aroma Organization

To assess variation in aroma composition within categories, we computed pairwise similarity scores among the KFO composition of aroma profiles within each category (Figure 1d; see Methods). Lower within‐category similarity scores indicate higher heterogeneity among KFO compositions, whereas higher scores reflect convergence toward shared compositions. Plant‐derived categories, including vegetables, fruits, juices and nectars, and coffee, cocoa and tea, exhibited the lowest similarity. In contrast, animal‐derived categories showed higher similarity, despite often containing larger numbers of KFOs (Table S2).

Hierarchical clustering based on cross‐category KFO composition similarity further organized aromas into distinct groups (Figure 1c). Cluster 1 comprised categories associated with either diverse biosynthetic origins or thermally and lipid‐driven chemistry. Within this cluster, plant‐based categories occupied a broad region of the aroma space, accompanied by a broad distribution of alcohols, aldehydes, and other biosynthetically derived structural classes (Figure S1). By comparison, animal‐derived foods formed tight subclusters characterized by highly similar KFO compositions.

Cluster 2 comprised food categories with relatively more similar KFO compositions. Water‐based beverages contained the fewest KFOs and showed high within‐category similarity. Alcoholic beverages, although comprising many fermentation‐derived KFOs, also exhibited strong convergence across samples. Milk and dairy products, sugar and confectionery products likewise showed high similarity, yielding stable chemical profiles despite differences in raw materials and processing (Figure S1). These clusters highlight marked differences in compositional heterogeneity, with some exhibiting highly diverse KFO profiles, whereas others show a relatively constrained and similar set of key odorants.

### 2.3 Generalist Key Odorants Stabilize Aroma Identity, Whereas Intermediaries Drive Diversity

**FIGURE 2:**
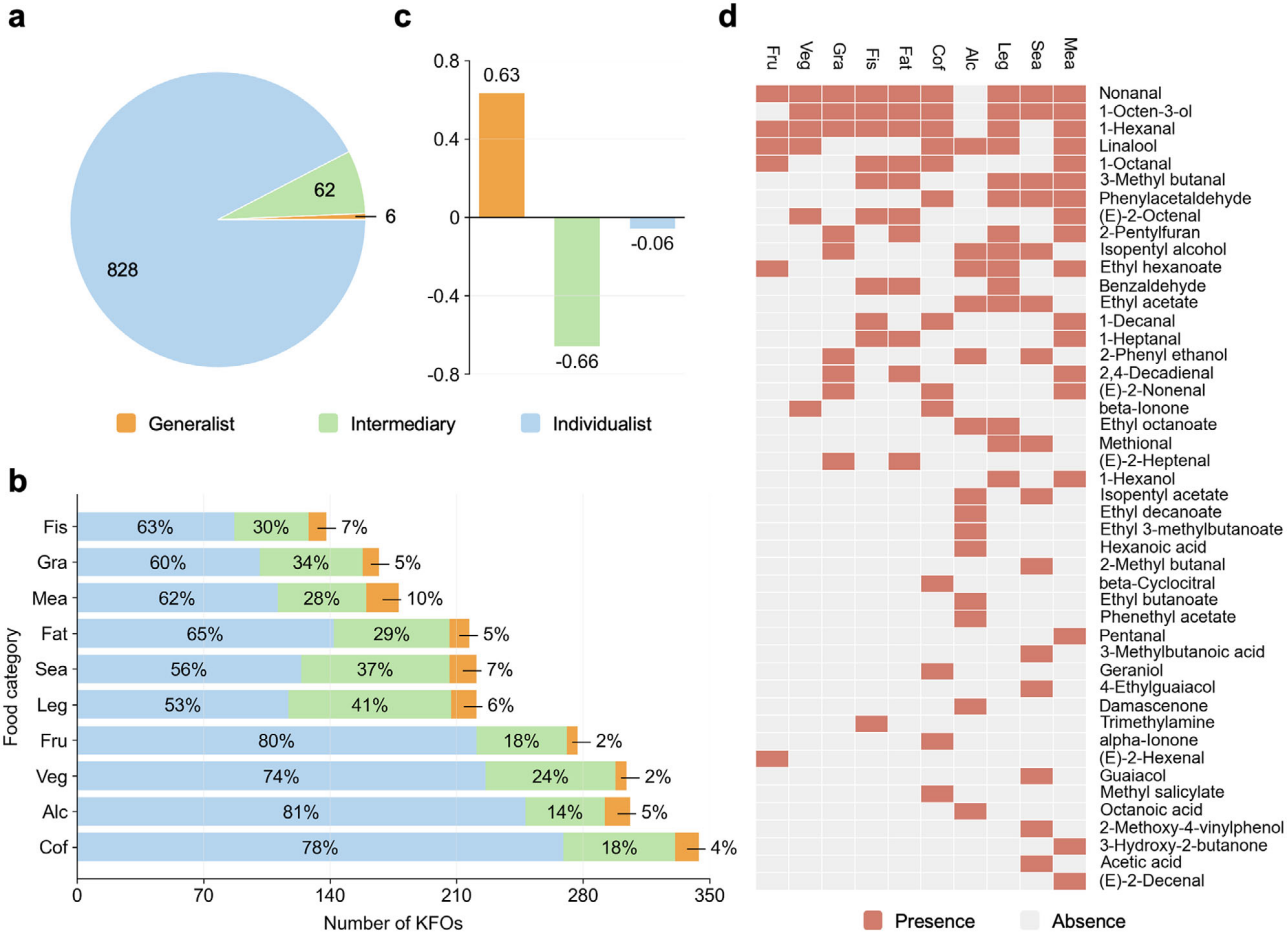
Frequency‐based key odorant classes play complementary roles in aroma organization. (a) Distribution of 896 KFOs classified into three frequency‐based groups across 2,282 food aroma profiles: generalists (>25%), intermediaries (5%–25%), and individualists (<5%). (b) Counts of generalists, intermediaries, and individualists identified within each of the ten balanced categories analyzed in this study. (c) Correlations between the abundance of each KFO frequency group within a food category and its within‐category compositional similarity score. Pearson correlation coefficients (r) are shown for each group. (d) Presence‐absence matrix of all generalist KFOs across categories, illustrating their distribution across categories and the occurrence of category‐specific generalists. Note: In panels (b) and (d), food category abbreviations are as follows: fis, fish and seafood; gra, grain products; mea, meat products; fat, fats and oils; sea, seasonings and condiments; leg, legumes, nuts, oilseeds and spices; fru, fruit products; veg, vegetable products; alc, alcoholic beverages; cof, coffee, cocoa and tea.

To characterize how key odorants are distributed across foods, we classified all 896 KFOs based on their frequency across food aroma profiles into generalists (present in >25% of samples), intermediaries (5%–25%), and individualists (<5%) following the previous approach [7] (Figure 2a). Only six compounds—2‐phenylethanol, linalool, 1‐octen‐3‐ol, ethyl hexanoate, 1‐hexanal, and nonanal—qualified as generalists. Intermediaries (n = 62) showed moderate prevalence and typically arise from pathway activations tied to botanical origin, fermentation, or thermal processing. The vast majority (828; 92.4%) were individualists, including 164 odorants found only once.

This frequency hierarchy was structured by chemical groups (Figure S2). Generalists were largely restricted to alcohols, aldehydes, and esters, whereas intermediaries spanned a broader range of structural classes. Individualists encompassed nearly all chemical groups, including several classes that were exclusively observed in this category, such as lactones, nitriles/amides, acetals, and halogens.

To examine how frequency‐based roles differ within individual aroma categories, we profiled KFO frequency across 10 balanced, data‐rich categories (Table S3; see Methods), thereby minimizing biases arising from uneven category representation. The relative proportions of generalists, intermediaries, and individualists varied markedly across categories (Figure 2b). Meat aroma profiles contained the largest number of category‐level generalists, whereas plant‐derived categories generally exhibited fewer generalists. These patterns parallel our earlier observations that plant categories exhibit higher diversity of KFO compositions, whereas those of animal‐based groups are more constrained and similar. Several categories also displayed category‐specific generalists (Figure 2d). Alcoholic beverages showed the highest proportion of exclusive generalists (50%), such as ethyl decanoate, ethyl 3‐methylbutanoate, and hexanoic acid, followed by seasonings and condiments (47%). In contrast, vegetables, legumes, nuts, spices, and fats and oils showed no exclusive generalists.

Correlation analysis further clarified the functional roles of these frequency classes (Figure 2c). The number of generalists in a category was positively associated with within‐category similarity (r = 0.63, p = 0.05), suggesting that these widely shared odorants form a backbone that stabilizes aroma identity. Intermediaries showed a strong negative correlation (r = −0.66, p = 0.04), indicating they drive divergence, whereas individualists showed no significant association (r = −0.06, p = 0.88), consistent with their role in encoding product‐specific nuance.

### 2.4 Distinct Foods Can Converge on Similar KFO Compositions

**FIGURE 3:**
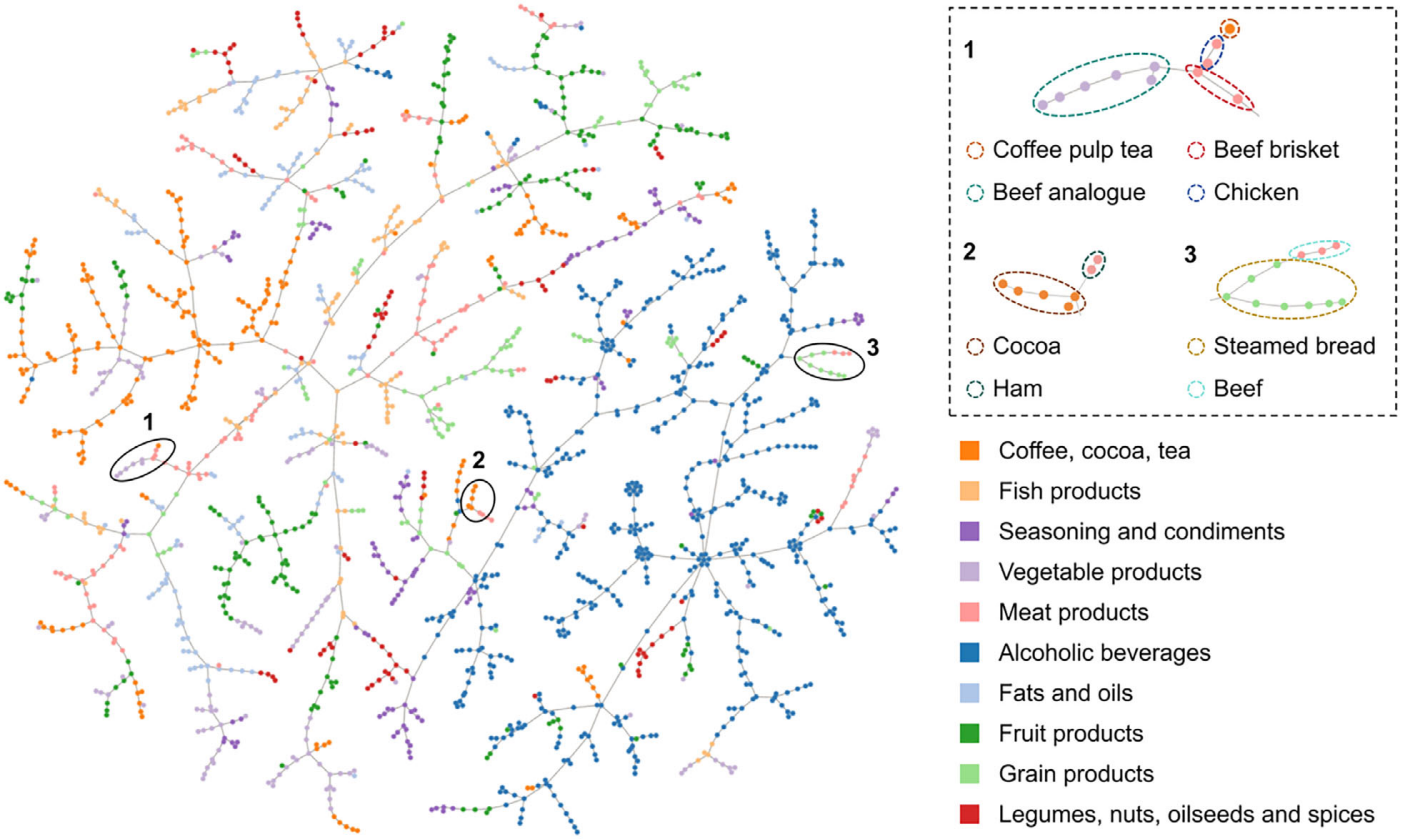
Cross‐category convergence of key odorant compositions across foods. 2D space of 1,876 aroma profiles from 10 major categories based on key food odorants (KFO) composition, in which each node represents an aroma profile. Nodes connected by edges and located in close proximity represent aroma profiles with similar KFO compositions. Different categories exhibit distinct spatial organizations: for example, alcoholic beverages form a densely clustered region, whereas legumes, nuts, oilseeds, and spices occupy a highly dispersed region of the map. Despite overall category separation, several annotated cross‐category neighborhoods are observed. In the plot, we annotated Groups 1, 2, and 3. Group 1 includes aroma profiles of plant‐based meat analogues (light violet dots) and coffee pulp tea (orange dots) positioned adjacent to those of beef brisket and roasted chicken (pink dots). Group 2 shows selected cocoa products located near ham profiles. Group 3 places fermented Chinese steamed bread (green dots) in close proximity to beef profiles (pink dots).

To map compositional similarity among individual food aroma profiles in the KFO‐Atlas, we projected them into a 2D space using TMAP [17], a tree‐based dimensionality reduction method, in which each node represents an aroma profile and connected local neighborhoods reflect similarity in KFO composition (Figure 3; see Methods). The resulting map showed pronounced differences in spatial distribution across food categories. Alcoholic beverages formed a densely clustered region, whereas legumes, nuts, oilseeds, and spices occupied a highly dispersed region in the space.

Despite overall category‐level separation, multiple cross‐category neighborhoods were observed in the aroma space (Figure 3). In Group 1, coffee pulp tea and plant‐based meat analogues were positioned adjacent to beef brisket and roasted chicken because they shared several lipid‐oxidation‐derived odorants [18, 19, 20, 21]. In Group 2, special cocoa products and ham clustered together due to shared Maillard‐ or lipid‐oxidation odorants [22, 23]. Group 3 revealed proximity between beef and a fermented Chinese steamed bread. These aroma profiles shared multiple odorants, including butanoic acid, styrene, 3‐hydroxy‐2‐butanone, and benzothiazole [24, 25, 26]. The presence of butanoic acid, which is commonly associated with animal fats, within sourdough systems is potentially due to metabolic cross‐talk between lactic acid bacteria and yeasts during fermentation, demonstrating the capacity of cereal‐based fermentations to generate “meaty” olfactory notes.

Taken together, the analysis identifies instances in which aroma profiles from different categories occupy adjacent regions of the space and share overlapping sets of KFOs. This convergence offers an explanation for why real‐world matrices with different biological origins or culinary treatments may nonetheless exhibit related aromatic signatures. It also suggests the possibility of mimicking the aroma signatures of animal meat by assembling odorants found in plant‐derived foods, highlighting an opportunity to reduce dependence on resource‐intensive livestock systems.

### 2.5 KFO‐Gen Generates Perceptually Valid Aroma Formulations

**FIGURE 4:**
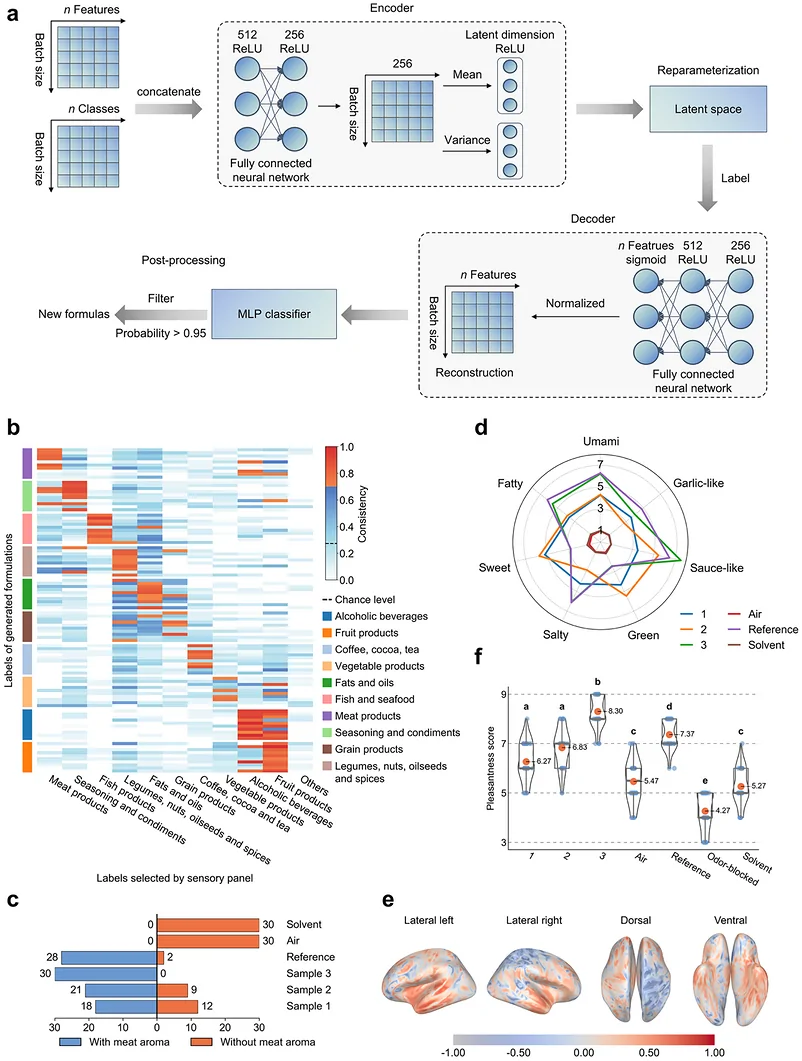
KFO‐Gen generates and validates perceptually valid aroma formulations. (a) Schematic of the conditional variational autoencoder used to generate KFO formulations targeting predefined aroma categories, with outputs further filtered by a multilayer perceptron classifier for category specificity. (b) Sensory evaluation of 100 AI‐generated formulations, showing the proportion of samples assigned by assessors to their intended aroma categories (*n* = 30 assessors) and consistency between AI and human assessors. (c) Sensory classification results for three AI‐generated, plant‐derived formulations targeting “meat”, with the count of assessors identifying each formulation as meat‐like (*n* = 30). (d) Sensory attribute profiles of different samples across multiple aroma descriptors (*n* = 30). (e) Electroencephalography source localization analysis comparing neural activation patterns elicited by sample 3 and the literature‐derived meat aroma reference, highlighting overlapping activated regions (*n* = 8). Colors indicate the correlation scores. (f) Pleasantness ratings (1‐9) obtained in an orthonasal‐blocked tasting experiment using plant‐based sausages, comparing samples with retronasal aroma enabled (*n* = 30). Note: In panel (f), blue and orange scatter points represent individual pleasantness ratings assigned by participants. Gray box plots summarize the data distribution for each sample, indicating the first and third quartiles, the median, and the range of non‐outlying values. Dark red points denote the mean pleasantness for each sample, and black curves represent the corresponding probability density distributions.

To explore whether aroma formulations can be computationally generated, we developed KFO‐Gen, a generative framework based on a conditional variational autoencoder (CVAE) trained to learn the latent structure of KFO combinations and their relative proportions (Figure 4a, see Methods). Coupled with a multilayer perceptron classifier for post‐hoc control, the model enables the generation of formulations targeted to predefined aroma categories. The CVAE demonstrated stable convergence (validation loss = 0.07; Figures S3 and S4 and Tables S4 and S5) and outperformed generative adversarial networks and conditional denoising diffusion probabilistic models in this task (see Supporting Information). The multilayer perceptron classifier also achieved robust predictive performance (ROC‐AUC = 0.96, mean average precision = 0.85; Figure S5).

When generating 1,000 candidate formulations per category under a 5–20 KFO constraint, 853 high‐confidence outputs (classifier probability > 0.95) were obtained. The average maximum similarity between generated and existing formulations within the target category was 0.58, indicating the ability to generate novel combinations (Table S6). To assess perceptual validity, 30 experienced panelists evaluated 100 randomly selected AI‐generated formulations (10 per major category) in a blinded setting (Data S2). Across categories, 73% of generated formulations were assigned by panelists to their intended aroma category (Figure 4b), significantly exceeding the chance‐level expectation (*p* < 0.01, see *Methods*).

### 2.6 KFO‐Gen Reconstructs Meat‐Like Aroma from Plant‐derived Odorants

Inspired by the cross‐category convergence observed earlier, we next tested whether the model could recreate meat‐like aromas under a source‐constrained design scenario, in which only KFOs previously reported in plant‐derived food formulations were allowed. Among 730 generated candidates, three formulations composed exclusively of plant‐derived KFOs were randomly selected for sensory evaluation (Table S7). Two formulations were classified as meat‐like by 65–70% of assessors, whereas one formulation (sample 3) was classified as meat‐like by all assessors (Figure 4c). Duplicate evaluations showed no significant differences between repeated measurements (paired‐sample t‐tests; Figure S6). Sensory profiling further showed that sample 3 exhibited attribute intensities similar to the aroma profile of the literature‐derived meat aroma reference [27] across multiple descriptors (Figure 4d), while relying on a much reduced number of KFOs derived from plant‐based sources (Table S7).

To further compare neural responses elicited by sample 3 and the literature‐derived meat aroma reference [27], EEG source localization analysis was performed. Compared with the correlation pattern obtained between the air condition (no odor stimulation) and the literature‐derived meat aroma reference (Figure S7), the AI‐generated sample 3 showed stronger and more spatially consistent correlations with the literature‐derived meat aroma reference across cortical regions. The highest regional correlations were observed across multiple brain regions (Figure 4e), including the parahippocampal gyrus (r = 0.46), insula (r = 0.45), superior temporal gyrus (r = 0.43), and posterior cingulate cortex (r = 0.43) [28].

To evaluate the contribution of the designed aroma formulation to pleasantness, an orthonasal‐blocked tasting experiment was conducted using plant‐based sausages. Allowing retronasal aroma resulted in higher pleasantness scores compared to aroma‐blocked conditions, showing the importance of aroma enhancement for plant‐based meat alternatives (Figure 4f). Among the evaluated aromas, sample 3 had the highest pleasantness score and achieved a higher pleasantness rating than the literature‐derived reconstituted meat aroma reference under the same experimental conditions [27].

## 3 Discussion

Understanding how complex odorant mixtures encode stable aroma identities is important for both basic olfactory science and rational aroma design. Here we provide KFO‐Atlas, a curated atlas of 896 KFOs across 2,282 aroma profiles, and identify recurrent statistical features of aroma organization, including sparsity, frequency‐based associations, and cross‐category compositional convergence, that structure real‐world aroma identities.

Analysis of KFO‐Atlas revealed a structured hierarchy in which generalists, intermediaries, and individualists play complementary functional roles. Generalists form a shared backbone that stabilizes aroma identity, intermediaries drive divergence, and individualists capture product‐specific nuances. Together, this hierarchy indicates that aroma is structured and interpretable rather than arbitrary. The thresholds used to define generalist, intermediary, and individualist KFOs were adopted from previous studies to preserve comparability [7]. These categories should therefore be regarded as operational rather than fixed boundaries. As larger and more harmonized KFO datasets become available, future studies may re‐evaluate whether alternative or data‐driven thresholds provide a more informative classification of KFO prevalence.

These organizational rules vary across mixture classes. In KFO‐Atlas, plant‐origin profiles shaped by diverse biosynthetic routes show highly heterogeneous odorant compositions. In contrast, lipid‐rich and thermally processed profiles (e.g., many meat products) repeatedly converge on odorants associated with lipid oxidation and Maillard chemistry, resulting in more constrained and similar compositions. Importantly, cross‐category neighborhoods in KFO‐Atlas show that chemically distinct mixtures can, in specific cases, assemble similar odorant combinations through shared reaction pathways. This convergence is consistent with shared biochemical reaction pathways and provides a plausible compositional interpretation for sensory similarities between certain foods, although direct causal mechanisms remain to be experimentally tested.

This structural convergence has direct implications for aroma design. Aroma categories governed by constrained chemical compositions occupy relatively limited regions of the KFO space, rendering their aroma profiles more learnable and reconstructable. Meat products exemplify this pattern: despite diversity in raw materials and processing conditions, their aromas consistently converge on a limited set of odorants.

Building on this structural property, we developed KFO‐Gen to learn the latent organization of KFO combinations and generate category‐targeted aroma formulations. Blinded sensory evaluation showed that most AI‐generated formulations were perceptually recognized as their intended categories, indicating that the model captures perceptually meaningful organization rather than merely compositional similarity. This advances beyond conventional AI approaches focused on individual odorants, addressing a longstanding challenge in modeling the emergent sensory properties of multi‐odorant mixtures. In this respect, our results complement recent efforts in odorant perception prediction and mixture modeling [29] by demonstrating that perceptually coherent mixture organization can be learned and validated at the level of real‐world odorant combinations.

Using exclusively plant‐derived KFOs, the model generated formulations that were consistently perceived as meat‐like by human assessors. Notably, assessment showed that one plant‐derived formulation elicited EEG response patterns more similar to those induced by a literature‐derived reconstituted meat aroma reference than to those observed under the air‐control condition. This finding provides complementary physiological support consistent with perceptual similarity between the generated formulation and the meat aroma reference. Beyond categorical recognition, the generated aroma formulations also influenced consumer‐relevant hedonic perception during food consumption. Allowing retronasal aroma substantially increased pleasantness ratings of plant‐based sausages, and the best‐performing plant‐derived aroma formulation exceeded the pleasantness of the literature‐derived reconstituted meat aroma reference. These findings indicate that generative aroma design can not only reproduce established sensory profiles but also access regions of aroma space that may remain underexplored in conventional formulation strategies.

Several limitations point to directions for future development. Although KFO‐Atlas substantially expands the reported landscape of KFOs, it remains a literature‐derived atlas assembled from studies conducted by different laboratories under heterogeneous experimental conditions. Differences in sample preparation and extraction methods, analytical platforms, quantification strategies, and odor threshold sources may introduce variability in KFO identification and OAV estimation and may affect different food categories unevenly. Accordingly, absolute quantitative comparisons across studies should be interpreted with caution. However, the principal analyses in this study rely primarily on recurrent compositional patterns observed across a large number of independently reported profiles rather than on direct cross‐study comparison of absolute concentrations. KFO‐Atlas should therefore be interpreted as a standardized integration of literature‐reported key odorants instead of a fully harmonized quantitative dataset. The generative model is likewise constrained by data availability, as categories represented by fewer aroma profiles may yield less stable latent representations and, consequently, less robust outputs. The validation of the generated formulations should also be interpreted within the scope of the present study. Here, sensory evaluation and EEG analysis were used to determine whether chemically defined, model‐generated aroma formulations were perceptually recognizable and physiologically consistent with their intended aroma categories. Future studies could complement this validation with headspace GC‐MS/O or GC×GC analyses to verify formulation preparation, volatile release, chemical stability, and matrix‐dependent partitioning, particularly after incorporation into real food matrices where these factors may alter the perceived aroma composition. Olfactory receptor assays could further provide mechanistic insight into receptor‐level activation by the generated formulations.

In addition, the FoodEx2 [16] classification system, while comprehensive, was developed for regulatory and compositional purposes rather than for sensory or perceptual analysis. Future work would benefit from aroma‐ or odor‐oriented ontologies that encode perceptual relationships between mixtures. Beyond ontology and data expansion, the present framework can be extended to incorporate practical constraints, such as ingredient availability, cost, and manufacturing feasibility, thereby moving from conceptual generative aroma design toward broader applications.

KFO‐Atlas and KFO‐Gen together provide complementary resources for understanding and modeling real‐world odorant mixtures. At a fundamental level, they offer system‐level insight into how combinations of odor‐active molecules are organized and interact to give rise to stable perceptual identities. At an applied level, food aroma provides a societally relevant test case for these principles. Off‐notes are a major obstacle to the development of sustainable food alternatives [30, 31, 32]. This framework offers a potential route to addressing this problem by enabling more rational aroma formulation, accelerated prototyping, and the development of plant‐based protein products that capture key sensory attributes traditionally associated with animal‐derived foods, thereby supporting a transition toward food systems that reduce reliance on livestock and its associated environmental burdens.

KFO‐Atlas and KFO‐Gen establish a foundation for investigating real‐world aromas arising from complex mixtures. By revealing when and why perceptual identities become reconstructable from combinations of key odorants, this work enables a more predictive approach to studying olfaction and chemical mixture perception, with food aroma serving as a scalable model system rather than a limiting application domain.

## 4 Methods

### 4.1 Construction and Curation of KFO‐Atlas

Here, we refer to literature‐derived KFO sets as aroma profiles and to model‐generated mixtures as aroma formulations. The term KFOs has been applied using slightly different operational criteria across the food aroma literature, reflecting differences in analytical objectives and experimental validation. Because the aim of this study was to construct a standardized, literature‐scale atlas of food aroma profiles spanning a large diversity of food matrices, we adopted a unified operational definition to enable consistent annotation across studies. Specifically, KFOs were defined as volatile compounds with OAVs greater than one, a criterion widely used in contemporary food aroma research [6]. To assemble a comprehensive and up‐to‐date dataset of KFOs, we conducted a structured literature survey of food aroma studies published between January 2015 and July 2024. This approximately ten‐year window provides broad coverage of recent food aroma research while reducing methodological heterogeneity, as advances in instrumental and sensory analysis have improved the consistency of KFO characterization.

Publications were retrieved from Google Scholar using a Boolean keyword strategy (“food” AND “aroma” AND “GC” AND “OAV”), yielding 5,892 candidate records. Titles and abstracts were initially screened using FoodBERT [33], a domain‐specific language model trained to identify food‐related content, to remove clearly irrelevant studies reporting non‐food systems or lacking aroma analysis. All remaining records were subsequently examined manually to determine whether they reported specific food items or ingredients, along with quantitative aroma information suitable for KFO annotation. Studies were retained if they reported identifiable food matrices and volatile compounds with odor activity values or sufficient quantitative information to identify KFOs. Records were excluded if they were reviews, method‐only papers, studies of non‐food systems, studies lacking OAV‐based or quantitative aroma information, or studies in which the food matrix or odorant identities could not be unambiguously extracted. After this two‐stage filtering, 602 publications report specific food items or ingredients and quantitative aroma analysis, and therefore were retained for data extraction.

For each eligible study, KFOs were manually curated and cross‐validated. Extracted information included food identity, lists of KFOs detected under the reported experimental conditions, corresponding OAVs, odor thresholds, and quantitative concentrations when available. To ensure consistency across studies, all compounds were standardized using the PubChem ID identifier. As OAVs reported in the literature vary substantially due to differences in food samples and experimental conditions, we retained the OAVs as reported in the original studies, without attempting cross‐study re‐standardization. Accordingly, KFOs were consistently identified based on a relative criterion (OAV>1) within each study, reflecting compounds perceived as aroma‐active under their specific food sample and experimental context. For analyses requiring proportional KFO composition vectors, the proportions were calculated from the reported quantitative concentrations. Specifically, after KFOs were identified according to the OAV > 1 criterion, the reported concentration of each KFO within an aroma profile was divided by the summed concentration of all KFOs in that profile. Each vector element therefore represents the relative concentration contribution of a given KFO, and all elements in each profile sum to one.

Each aroma profile was assigned to a food category using the FoodEx2 [16] classification system, resulting in 20 major food categories and 86 subcategories. This hierarchical annotation enabled consistent comparison across diverse food systems. For example, meat products were further resolved into mammal meat, bird meat, dried meat, cooked cured meat, raw cured meat, and preserved sausages. Food items that could not be unambiguously mapped to an existing subcategory (for example, composite fermented cereal‐dairy products such as Tarhana) were assigned at the major‐category level only to avoid introducing artificial classification bias.

To characterize how different odorants contribute to food aroma organization, KFOs were classified according to their occurrence frequency across aroma profiles, following and extending a previously established framework [7]. Compounds detected in more than 25% of aroma profiles were designated generalists, those occurring in 5–25% were defined as intermediaries, and those present in fewer than 5% of aroma profiles were classified as individualists.

All KFOs were additionally assigned to one of 18 chemical classes based on molecular structure, using the Volatile Compounds in Food reference classification (https://www.vcf‐online.nl/VcfCompounds.cfm). These classes included alcohols, aldehydes, ketones, esters, acids, furans, phenols, sulfur compounds, hydrocarbons, bases, lactones, (ep)oxides/pyrans/coumarins, ethers, acetals, nitriles/amides, oxazol(in)es, halogens, and imidazoles (Table S1).

### 4.2 Clustering of Aroma Profiles

To characterize the organizational structure of aroma compositions across foods, we restricted hierarchical analyses to food categories represented by at least ten aroma profiles, ensuring sufficient sampling to yield stable similarity estimates. This filtering resulted in a curated dataset of 2,274 aroma profiles for comparative analysis. Compositional relationships among profiles were visualized using hierarchical clustering implemented with PyComplexHeatmap [34]. Clustering was performed on proportional KFO composition vectors using correlation distance and average linkage. Correlation distance was selected to emphasize similarities in relative odorant patterns rather than absolute KFO abundance, allowing aroma profiles with comparable compositional structure to cluster together despite differences in scale.

### 4.3 Similarity Calculation between Aroma Profiles

To quantify compositional similarity between aroma profiles, we defined a compositional similarity score as the arithmetic mean of cosine similarity and a normalized Manhattan‐distance‐derived similarity (Equation 1). Specifically, we integrated cosine similarity, which reflects directional similarity in KFO composition, with Manhattan distance, which captures absolute proportional deviations between aroma profiles. For two aroma profiles*A_i_* and *B_i_*, all KFOs identified across the dataset were first expanded into a unified feature vector, with absent compounds assigned a value of zero. Each vector element represents the proportional contribution of a given KFO within the aroma profile. Similarity between aroma profiles was then computed as:

(1) $$\begin{array}{ccc} \mathrm{Similarity} & = & w\times \frac{\sum_{i=1}^{n}A_{i}B_{i}}{\sqrt{\sum_{i=1}^{n}A_{i}^{2}}\cdot \sqrt{\sum_{i=1}^{n}B_{i}^{2}}}+\left(1-w\right)\\ & & \times \;\left(1-\frac{\sum_{i=1}^{n}\left|A_{i}-B_{i}\right|}{2}\right) \end{array}$$

In this study, w = 0.50 was used, corresponding to equal weighting of cosine similarity and normalized Manhattan similarity (see Figure S3). The resulting similarity scores range from 0 to 1, with higher values indicating greater overlap in KFO composition.

For each food category, within‐category similarity was defined as the mean of all pairwise similarities among aroma profiles belonging to that category. This provides a robust estimate of category‐level aromatic homogeneity while accounting for differences in both KFO identity and relative abundance.

### 4.4 Hierarchical Roles of Key Odorants in Shaping Aroma

To resolve category‐specific frequency patterns while ensuring statistical robustness, we restricted the analysis to food categories represented by at least 50 aroma profiles, and to subcategories with at least 20 aroma profiles. This filtering yielded a balanced, data‐rich subset comprising ten well‐represented food categories (Table S3). Aroma profiles corresponding to composite dishes were excluded because they integrate multiple ingredient types and confound category‐level interpretation. In addition, fruit and vegetable juices and nectars were removed owing to their substantial raw‐material overlap with whole fruit and vegetable categories in the FoodEx2 [16] classification.

Within this curated dataset, KFOs were classified as generalists, intermediaries, or individualists based on their occurrence frequency, and the abundance of each class was quantified at the category level. To assess how different KFO frequency classes relate to category‐level aroma organization, we computed Pearson correlations between the number of generalist, intermediary, and individualist KFOs in each category and the corresponding within‐category similarity scores. This analysis quantifies the extent to which different classes of KFOs contribute to aromatic convergence or heterogeneity across food categories.

### 4.5 Cross‐Category Convergence Analysis

To examine cross‐category convergence among aroma profiles, we constructed a global aroma space across the ten balanced, data‐rich food categories (Table S3) using TMAP [17], a tree‐based dimensionality‐reduction method optimized for sparse data. TMAP preserves local neighborhood relationships, making it well‐suited for identifying convergent molecular architectures among complex odorant mixtures.

For each aroma profile, the proportional contributions of all KFOs were scaled to a uniform range and discretized into 8‐bit unsigned integer vectors, satisfying the input requirements of the TMAP hashing pipeline while preserving relative compositional information. The resulting vectors were embedded in a tree‐structured map that encompassed all eligible aroma profiles. Nodes were colored by food category, and representative products were annotated to facilitate interpretation. In this global aroma map, tightly packed branches reflect strong compositional convergence within food categories. In contrast, mixed branches indicate cross‐category molecular overlap, highlighting instances where distinct food matrices converge on similar odorant architectures.

### 4.6 Development of Supervised Machine Learning Models

To enable post hoc quality control of generated aroma formulations, we developed supervised classification models to predict food category membership based on KFO composition. Using the ten balanced, data‐rich food categories, together with an additional ‘others’ class representing chemically plausible but category‐agnostic formulations (Table S3), we benchmarked multiple classifiers, including linear discriminant analysis, decision trees, support vector machines, logistic regression, and multilayer perceptron. The ‘others’ class was constructed from randomly generated, category‐agnostic sparse KFO composition vectors (see Supporting Information). Briefly, each vector contained a random number of KFOs within the empirical range of profile sizes, with KFO identities and weights randomly assigned and normalized to sum to one. To reduce overlap with empirical aroma profiles, only vectors with an average similarity below 0.2 to all empirical formulations were retained. Formulations were represented by their proportional KFO composition vectors and labeled according to food categories, with the ‘others’ class introduced to capture off‐category formulations and improve robustness. Model training and evaluation were performed using stratified individual‐profile‐level splitting. This strategy was selected because the cross‐food‐category dataset was imbalanced across categories and source studies, and stratification at the profile level preserved category representation in both training and testing sets. Among the tested approaches, the multilayer perceptron achieved the best overall performance (macro F1 = 0.87 ± 0.02; Table S8) and was therefore selected as a probabilistic filter to screen CVAE‐generated formulations, ensuring category fidelity in downstream analyses. The classifier was not used as an evaluation metric for the generative model but only as a post hoc filtering step to remove clearly off‐category candidates. Full benchmarking details are provided in the Supporting Information.

### 4.7 Development of KFO‐Gen Based on Conditional Variational Autoencoder

To enable de novo generation of aroma formulations targeted to specific food categories, we developed KFO‐Gen, a CVAE‐based generative framework integrated with a supervised classifier for post hoc quality control. Its performance was benchmarked against conditional generative adversarial networks and conditional denoising diffusion probabilistic models (see Supporting Information). The model was trained on formulations from ten balanced, data‐rich food categories, together with an additional ‘others’ class representing chemically plausible but category‐agnostic formulations. Input features consisted of proportional KFO composition vectors. The CVAE encoder jointly processes the KFO composition vector and the corresponding category label to learn a latent representation that captures category‐conditioned structure in KFO combinations. The encoder outputs the mean (µ) and log‐variance (log σ^2^) parameters of a multivariate Gaussian latent distribution, from which latent variables are sampled using the reparameterization trick. The decoder receives the sampled latent vector and the category label, and reconstructs a formulation vector using multilayer perceptrons with ReLU activations, followed by a sigmoid output layer that constrains all values to the [0,1] interval.

Because empirical aroma formulations are sparse and dominated by a limited number of key odorants, we applied a sparsity‐preserving post‐processing step to all generated outputs. For each decoded formulation, only the top k KFOs (with k constrained between 5 and 20 during validation) were retained, and the resulting vectors were renormalized to sum to one. This procedure yields formulations that reflect the structure observed in real foods.

To improve category consistency of the generated formulations, we applied a multilayer perceptron classifier as a post hoc probabilistic filter. Only formulations assigned to the target category with high confidence (probability >0.95) were retained.

The CVAE was trained using a composite objective comprising a reconstruction term and a Kullback‐Leibler divergence regularization term. Reconstruction loss was computed using mean squared error between the input and reconstructed formulation vectors (Equation 2), whereas the Kullback‐Leibler term encouraged the latent space to approximate a standard normal prior (Equation 3). The total loss (Equation 4) was represented as:

(2) $$L_{recon}=\sum {\left(\hat{x}-x\right)}^{2}$$

(3) $$L_{KL}=-0.5\sum \left(1+\log\sigma^{2}-\mu^{2}-\sigma^{2}\right)$$

(4) $$L_{total}=w_{recon}L_{recon}+w_{KL}L_{KL}$$

The AdamW optimizer was used for parameter updates. A cosine annealing schedule was used to modulate β across epochs, while a ReduceLROnPlateau scheduler halved the learning rate when the validation loss plateaued (patience = 20 epochs). Gradient clipping (maximum norm = 1.0) stabilized training, and early stopping was triggered when validation loss failed to improve over the specified patience epochs. To identify an optimal architecture and training configuration, we conducted an extensive hyperparameter search (1,000 trials), with each trial capped at 1,000 epochs. The best‐performing model was selected based on minimum validation loss. Full hyperparameter ranges are summarized in Table S5.

To quantify the novelty of generated formulations relative to existing data, we calculated a average maximum similarity metric. For each generated formulation, its highest similarity to any formulation within the target category was identified and averaged across samples. Lower similarity scores indicate exploration of novel yet category‐consistent regions of the aroma formulation space.

All original data and code are publicly available following the key initiatives to advance AI in food science by Zhang et al. [35].

### 4.8 Sensory and Electroencephalography Assessment

Thirty experienced panelists were recruited for sensory evaluation. All participants were right‐handed, reported no respiratory, neurological, or olfactory disorders, and had no history of substance abuse. The study was conducted in accordance with institutional ethical guidelines and was approved by the Ethics Committee of the Shanghai Institute of Technology (approval number: SIT‐2024‐LL26).

Sensory evaluation comprised two sections (see Supporting Information). Section 1 evaluated the perceptual category fidelity of AI‐generated aroma formulations using a blinded category‐recognition task. A total of 100 AI‐generated formulations (10 randomly selected formulations from each of the ten food categories) were presented to participants, who judged whether each formulation matched its intended aroma category. Participants were allowed to select up to three perceived categories because aroma categories are not always perceptually exclusive. For example, some formulations may reasonably evoke both grape and wine aromas, or share perceptual characteristics among fruit products, juices, and nectars, reflecting naturally overlapping odorant compositions. Participants selected one to three perceived categories from 11 predefined response options, consisting of ten food categories and an additional ‘others’ option. A formulation was considered correctly recognized when its intended category was selected. Statistical significance above chance was assessed using a one‐sided exact binomial test. Because participants were allowed to select up to three of the 11 response options, we used 3/11 (27.3%) as a conservative upper‐bound estimate of the recognition probability expected under random guessing.

Section 2 focused specifically on meat‐targeted formulations generated exclusively from plant‐derived KFOs. This section comprised two complementary sensory evaluations. First, participants determined whether each aroma sample was perceived as having a meat aroma or not, after which they characterized the perceived aroma using an eight‐dimension aroma wheel. Second, the same aroma stimuli were presented while participants consumed a standardized plant‐based sausage to evaluate whether aroma exposure influenced hedonic perception during eating. Pleasantness was further compared between aroma‐available and aroma‐blocked conditions to assess the contribution of olfactory cues to food acceptance.

To complement sensory evaluation with physiological response measurements, EEG recordings (Figure S8) were obtained from eight participants during odor exposure to the AI‐generated Sample 3, reconstituted meat aroma reference, and air condition. Source localization and correlation analyses were used to compare neural activation patterns associated with aroma perception. Full experimental procedures and data processing pipelines for sensory and EEG analyses are provided in Supporting Information.

### 4.9 Statistical Analysis

Statistical procedures specific to data curation, compositional similarity analysis, machine‐learning evaluation, sensory evaluation, and EEG analysis are described in the corresponding Methods sections above. Statistical significance was evaluated at α = 0.05. Statistical analyses and visualization were performed using Python, including NumPy (2.1.3), pandas (2.3.2), SciPy (1.15.3), scikit‐learn (1.7.1), PyTorch (2.3.1), matplotlib (3.10.5), and seaborn (0.13.2).

## Author Contributions

**Jingzhi Zhang**: methodology, data curation, investigation, validation, visualization, writing – original draft, writing – review and editing. **Huadong Xing**: methodology, software. **Antonella Di Pizio**: methodology, conceptualization, writing – review and editing. **Qinfei Ke**: conceptualization, resources, data curation, investigation. **Xingran Kou**: conceptualization, resources, data curation, funding acquisition. **Dachuan Zhang**: conceptualization, writing – original draft, writing – review and editing, resources, supervision, project administration, visualization, methodology, software, validation, funding acquisition.

## Funding

This project was supported by the Collaborative Innovation Center of Fragrance Flavour and Cosmetics; Ministry of Education, Singapore, under the Academic Research Fund Tier 1 (A‐8004765‐00‐00); and NUS IT (NUSREC‐HPC‐00001).

## Conflicts of Interest

The authors declare no conflicts of interest.

## Supporting information




**Supporting File 1**: advs77740‐sup‐0001‐SuppMat.docx.

**Supporting File 2**: advs77740‐sup‐0002‐SuppMat.xlsx.

**Supporting File 3**: advs77740‐sup‐0003‐SuppMat.xlsx.

## Data Availability

KFO‐Atlas and KFO‐Gen are publicly available on Zenodo (https://zenodo.org/records/22291472 and https://zenodo.org/records/22291915). Other data generated in this research are available in supporting information files 2 and 3 (Data S1‐S2). Data on key food odorants were manually curated from publications retrieved via Google Scholar. Food classification data were collected from FoodEx2 (https://www.efsa.europa.eu/en/data/data‐standardization). Chemical group data of KFOs were collected from the Volatile Compounds in Food list (https://www.vcf‐online.nl/VcfCompounds.cfm). An interactive web interface for KFO‐Atlas is available at https://kfo‐atlas.foodmole.net/. It supports browsing and searching of KFO data and provides easy access to the KFO‐Gen aroma design model. The web interface is intended to remain available throughout the project period, whereas the complete KFO‐Atlas dataset is permanently archived on Zenodo to ensure long‐term accessibility.

## References

[1] M. S. Kehl , S. Mackay , K. Ohla , et al., “Single‐Neuron Representations of Odours in the Human Brain,” Nature 634, no. 8034 (2024): 626–634, 10.1038/s41586-024-08016-5. 39385026 PMC11485236

[2] P. A. Khorisantono , M. G. Veldhuizen , and J. Seubert , “Tastes and Retronasal Odours Evoke a Shared Flavour‐specific Neural Code in the Human Insula,” Nature Communications 16, no. 1 (2025): 8252, 10.1038/s41467-025-63803-6. PMC12432251 40940344

[3] Q. Ke , J. Zhang , X. Huang , X. Kou , and D. Zhang , “Machine Learning Unveils Three Layers of Food Complexity,” npj Science of Food 10 (2026): 87, 10.1038/s41538-026-00730-w. 41644539 PMC12988112

[4] D. Gopaulchan , C. Moore , N. Ali , et al., “A Defined Microbial Community Reproduces Attributes of Fine Flavour Chocolate Fermentation,” Nature Microbiology 10, no. 9 (2025): 2130–2152, 10.1038/s41564-025-02077-6. PMC12408344 40825855

[5] X. Kou , P. Shi , C. Gao , et al., “Data‐Driven Elucidation of Flavor Chemistry,” Journal of Agricultural and Food Chemistry 71, no. 18 (2023): 6789–6802, 10.1021/acs.jafc.3c00909. 37102791 PMC10176570

[6] Y. Yang , L. Ai , Z. Mu , et al., “Flavor Compounds With High Odor Activity Values (OAV >1) Dominate the Aroma of Aged Chinese Rice Wine (Huangjiu) by Molecular Association,” Food Chemistry 383 (2022): 132370, 10.1016/j.foodchem.2022.132370. 35183960

[7] A. Dunkel , M. Steinhaus , M. Kotthoff , et al., “Nature's Chemical Signatures in Human Olfaction: A Foodborne Perspective for Future Biotechnology,” Angewandte Chemie International Edition 53, no. 28 (2014): 7124–7143, 10.1002/anie.201309508. 24939725

[8] X. Song , M. E. Porter , V. M. Whitaker , S. Lee , and Y. Wang , “Identification of ethyl vanillin in strawberry (Fragaria × ananassa) using a targeted metabolomics strategy: From artificial to natural,” Food Chemistry: X 20 (2023): 100944, 10.1016/j.fochx.2023.100944. 38022735 PMC10663669

[9] T. Wongprasert , P. Mathatheeranan , P. Siripitakpong , et al., “Effect of Functional Groups in Strawberry Flavoring on Pea Protein‐flavor Interactions: Potential Applicable in Flavor Formulation for Plant‐based Protein Aqueous Foods,” Food Chemistry: X 23 (2024): 101702, 10.1016/j.fochx.2024.101702. 39184319 PMC11342753

[10] D. Zhang , “Practical Guide for Food Scientists to Build AI: Data, Algorithms, and Applications,” Food Chemistry 499 (2026): 147281, 10.1016/j.foodchem.2025.147281. 41325662

[11] B. K. Lee , E. J. Mayhew , B. Sanchez‐Lengeling , et al., “A Principal Odor Map Unifies Diverse Tasks in Olfactory Perception,” Science 381, no. 6661 (2023): 999–1006, 10.1126/science.ade4401. 37651511 PMC11898014

[12] J. Qian , X. Wang , F. Song , et al., “ChemSweet: An AI‐Driven Computational Platform for Next‐Gen Sweetener Discovery,” Food Chemistry 463 (2025): 141362, 10.1016/j.foodchem.2024.141362. 39326310

[13] P. Shi , X. Huang , Q. Ke , X. Kou , and D. Zhang , “Mapping Sleep‐Promoting Volatiles in Aromatic Plants with Machine Learning: A Comprehensive Survey of 2300 Molecules,” Digital Discovery 5, no. 3 (2026): 1068–1078, 10.1039/d5dd00173k.

[14] C.‐E. Tan , B. P. Neupane , Y. Wen , et al., “Volatile Organic Compound‐Based Predictive Modeling of Smoke Taint in Wine,” Journal of Agricultural and Food Chemistry 72, no. 14 (2024): 8060–8071, 10.1021/acs.jafc.3c07019. 38533667 PMC11010234

[15] S. Singh , D. Schicker , H. Haug , T. Sauerwald , and A. T. Grasskamp , “Odor Prediction of Whiskies Based on Their Molecular Composition,” Communications Chemistry 7, no. 1 (2024): 293, 10.1038/s42004-024-01373-2. 39702492 PMC11659623

[16] K. Niforou , N. Koffas , A. Livaniou , and S. Ioannidou , “FoodEx2 maintenance 2024,” *EFSA Supporting Publications* 22, no. 4 (2025): 9414E, 10.2903/sp.efsa.2025.EN-9414.

[17] D. Probst and J. L. Reymond , “Visualization of Very Large High‐Dimensional Data Sets as Minimum Spanning Trees,” Journal of Cheminformatics 12, no. 1 (2020): 12, 10.1186/s13321-020-0416-x. 33431043 PMC7015965

[18] D. Hu , G. Yang , X. Liu , et al., “Comparison of Different Drying Technologies for Coffee Pulp Tea: Changes in Color, Taste, Bioactive and Aroma Components,” LWT 200 (2024): 116193, 10.1016/j.lwt.2024.116193.

[19] Z. Zhang , M. Zang , J. Chen , et al., “Effect of the Mycelium of Oyster Mushrooms on the Physical and Flavor Properties of a Plant‐Based Beef Analogue,” LWT 198 (2024): 116029, 10.1016/j.lwt.2024.116029.

[20] Z. Zhang , M. Zang , K. Zhang , S. Wang , D. Li , and X. Li , “Effect of Two Types of Thermal Processing Methods on the Aroma and Taste Profiles of Three Commercial Plant‐Based Beef Analogues and Beef by GC‐MS, E‐nose, E‐tongue, and Sensory Evaluation,” Food Control 146 (2023): 109551, 10.1016/j.foodcont.2022.109551.

[21] Y. Wang , H. Zhang , K. Li , et al., “Dynamic Changes in the Water Distribution and Key Aroma Compounds of Roasted Chicken During Roasting,” Food Research International 172 (2023): 113146, 10.1016/j.foodres.2023.113146. 37689908

[22] Z. Xiao , L. Liu , Y. Niu , J. Zhang , D. Wang , and C. Zhou , “Mushroom Alcohol(1‐octen‐3‐ol)and Other 7 Aroma Compounds Selected From Chinese Dry‐Cured Hams Can Enhance Saltiness Perception,” Meat Science 208 (2024): 109398, 10.1016/j.meatsci.2023.109398. 38029506

[23] S. Collin , T. Fisette , A. Pinto , J. Souza , and H. Rogez , “Discriminating Aroma Compounds in Five Cocoa Bean Genotypes From Two Brazilian States: White Kerosene‐Like Catongo, Red Whisky‐Like FL89 (Bahia), Forasteros IMC67, PA121 and P7 (Pará),” Molecules 28 (2023): 1548. 36838536 10.3390/molecules28041548 PMC9961520

[24] J. Wan , Q. Liu , C. Ma , et al., “Characteristic Flavor Fingerprint Disclosure of Dzo Beef in Tibet by Applying SAFE‐GC‐O‐MS and HS‐GC‐IMS Technology,” Food Research International 166 (2023): 112581, 10.1016/j.foodres.2023.112581. 36914343

[25] Y. Huang , J. Wan , Z. Wang , et al., “Variation of Volatile Compounds and Corresponding Aroma Profiles in Chinese Steamed Bread by Various Yeast Species Fermented at Different Times,” Journal of Agricultural and Food Chemistry 70, no. 12 (2022): 3795–3806, 10.1021/acs.jafc.2c00550. 35294179

[26] Y. Huang , J. Wan , M. Sun , et al., “Flavor Profile Disclosure of Chinese Steamed Breads (CSBs) by sensomics Approach,” Food Bioscience 51 (2023): 102198, 10.1016/j.fbio.2022.102198.

[27] Z. Wang , T. Nie , Y. Gao , et al., “Decoding the Flavor of Traditional Chinese Stir‐fried Pork Cuisine: Development of a Sensory Wheel and Exploration of the Relationship Between Sensory Attributes and Aroma Compounds,” Food Research International 214 (2025): 116650, 10.1016/j.foodres.2025.116650. 40467236

[28] N. Kriegeskorte , M. Mur , and P. Bandettini , “Representational Similarity Analysis—Connecting the Branches of Systems Neuroscience,” Front Syst Neurosci 2 (2008): 4, 10.3389/neuro.06.004.2008. 19104670 PMC2605405

[29] V. Satarifard , L. Sisson , Y. Han , et al., “High‐Fidelity Tuning of Olfactory Mixture Distances in the Perceptual Space of Smell through a Community Effort,” BioRxiv (2025), 10.64898/2025.12.13.694160.

[30] B. Sharma , R. Keast , D. G. Liem , et al., “Plant‐protein Isolates and Flavour Perception: Understanding Mechanisms and Strategies to Balance Flavour Retention and Release,” Food Chemistry 493 (2025): 145815, 10.1016/j.foodchem.2025.145815. 40795561

[31] V. K. Mittermeier‐Klessinger , T. Hofmann , and C. Dawid , “Mitigating Off‐Flavors of Plant‐Based Proteins,” Journal of Agricultural and Food Chemistry 69, no. 32 (2021): 9202–9207, 10.1021/acs.jafc.1c03398. 34342446

[32] X. Yu , Z. Su , G. Xia , D. Zhang , H. Li , and W. Dong , “Elucidating the Interaction Between Pyrazine Flavor Compounds and Coffee Proteins: Insights From Multiscale Structural and Molecular Dynamics Simulations,” Current Research in Food Science 13 (2026): 101478, 10.1016/j.crfs.2026.101478. 42376624 PMC13311791

[33] C. Chambliss , “FBERT: Food Extraction with DistilBERT,” (2020), https://github.com/chambliss/foodbert.

[34] W. Ding , D. Goldberg , and W. Zhou , “PyComplexHeatmap: A Python Package to Visualize Multimodal Genomics Data,” Imeta 2, no. 3 (2023): 115, 10.1002/imt2.115. PMC10919210 38454967

[35] D. Zhang , M. Liu , Z. Yu , et al., “Domain Knowledge, Just Evaluation, and Robust Data Standards Are Required to Advance AI in Food science,” Trends in Food Science & Technology 164 (2025): 105272, 10.1016/j.tifs.2025.105272.

