# Supplementary material for: Molecular Atlas of Key Food Odorants Reveals Mixture‐Level Organization and Enables Generative Aroma Design
Source: Adv Sci (Weinh). 2026 Sep 13:e77740. Online ahead of print. doi: 10.1002/advs.77740 (PMC13572858; doi:10.1002/advs.77740)
Supplement: Supplementary file 1 [file ADVS-9999-e77740-s001.docx]

Supporting Information

**Molecular Atlas of Key Food Odorants Reveals Mixture-Level Organization and Enables Generative Aroma Design**

*Jingzhi Zhang, Huadong Xing, Antonella Di Pizio, Qinfei Ke***, Xingran Kou***, Dachuan Zhang**

**1. Development of Supervised Machine Learning Models**

Formulation vectors, including randomly generated samples assigned to the ‘others’ class, were encoded as proportional key food odorant (KFO) composition spanning all identified KFOs. Five supervised learning algorithms, including decision trees, support vector machines, logistic regression, linear discriminant analysis, and multilayer perceptron, were evaluated for classification performance.

Models were trained five times using five independent random seeds (100, 200, 300, 400, and 500). For each run, model evaluation was performed using a stratified split at the individual-profile level, which preserved food-category representation in the training and testing sets. This split level was used because the cross-food-category dataset was imbalanced across categories and source studies. Performance was assessed using weighted F1 scores and area under the ROC curve (ROC-AUC). Across all evaluated settings, the multilayer perceptron achieved the highest performance (AUC > 0.90 across categories; F1 score = 0.87; Figure S4; Table S7). This model was therefore selected for filtering CVAE-generated formulations.

## **2. Selection and Validation of the Compositional Similarity Metric**

**2.1. Rationale for the Similarity Metric**

Aroma profiles in KFO-Atlas were represented as compositional vectors, in which each element denotes the proportional contribution of an individual KFO and each vector is normalized to a constant sum of 100.00%. The similarity between two aroma profiles should therefore capture two complementary properties: whether the same KFOs dominate the profiles, and how strongly their relative proportions differ. Cosine similarity captures directional alignment between proportional vectors and is useful for identifying profiles dominated by similar sets of KFOs. However, cosine similarity alone is comparatively insensitive to absolute proportional displacement. In contrast, normalized Manhattan similarity captures absolute differences in component proportions but does not explicitly quantify directional alignment. We therefore used a composite similarity score defined as the equally weighted mean of cosine similarity and normalized Manhattan similarity.

For percentage-compositional vectors, the theoretical maximum L1 distance is 200.00. Thus, the Manhattan-derived similarity was calculated as 1-L1/200.00. This normalization is mathematically equivalent to Bray-Curtis similarity for vectors with a constant sum of 100.00%, meaning that the composite score integrates cosine similarity with a Bray-Curtis-equivalent abundance-sensitive component.

**2.2. Comparison with Alternative Similarity Metrics**

To evaluate whether the selected metric was robust to metric choice, we compared the composite score with cosine similarity, normalized Manhattan similarity, Bray-Curtis similarity, Jaccard similarity, Canberra similarity, and Euclidean similarity across the full KFO-Atlas dataset. Within-category similarity was calculated as the mean of all pairwise similarities among aroma profiles belonging to the same food category. Category-level homogeneity scores obtained with different metrics were then compared with Spearman’s rank correlation.

The composite cosine-Manhattan score was highly concordant with cosine similarity, normalized Manhattan similarity, Bray-Curtis similarity, Jaccard similarity, and Euclidean similarity, with category-level Spearman ρ values of 1.00, 0.99, 0.99, 0.97, and 0.91, respectively (Figure S3a). In contrast, Canberra similarity showed substantially weaker concordance with the composite score (Spearman ρ = 0.66). This discrepancy likely reflects the instability of Canberra distance for highly sparse compositional data, where near-zero denominators can amplify small differences across many low-abundance KFO dimensions.

Earth Mover’s Distance (EMD) requires a ground metric that describes the distance between KFO features, such as chemical or perceptual similarity. In the absence of such a feature-level ground metric, the default discrete-uniform cost matrix assigns zero cost to matching identical features and equal cost to transporting mass between any different features. Under this setting, EMD becomes mathematically equivalent to L1/2 for normalized probability distributions.

We verified this relationship using a stratified sample of 239 profiles, with up to 15 profiles from each food category. EMD and L1/2 agreed closely, with Pearson r=1.00, Spearman ρ=0.97, and identical mean values of 0.95 (Figure S3b). Therefore, without a chemically or perceptually meaningful KFO-level ground metric, EMD does not provide information beyond the normalized Manhattan component already included in the composite score.

**2.3. Sensitivity Analyses of the Combined Cosine-Manhattan Score**

To assess whether the equal-weight setting influenced category-level conclusions, we varied the cosine weight from 0.00 to 1.00 in increments of 0.10. For each value of w, we recalculated the within-category mean similarity for each food category, yielding smooth score trajectories across the weighting range (Figure S3c). We then compared the resulting category rankings with the baseline setting of w = 0.50 using Kendall’s τ (Figure S3d). Category rankings were identical to the baseline for w=0.20-0.60 (Kendall’s τ = 1.00) and remained highly similar even at the extreme settings (Figure S3d). The equal-weight setting was therefore retained because it lies in the center of a broad robust region and gives balanced importance to directional alignment and proportional displacement.

Together, these analyses support the use of the composite cosine-Manhattan similarity score for comparing KFO composition profiles. The metric is theoretically appropriate for normalized compositional data, robust to variations in weighting schemes, and stable under modest proportional perturbations. Compared with alternative metrics, Jaccard similarity discards abundance information, Canberra similarity is unstable for highly sparse compositional vectors, and EMD is redundant with L1 distance unless a meaningful KFO-level ground metric is introduced. We therefore used the equally weighted cosine-Manhattan similarity score for downstream analyses of aroma profile similarity, category-level homogeneity, and cross-category compositional convergence.

## **3. Generation of Formulations in the ‘Others’ Category**

To ensure the classification and generation models are not forced to assign one out of the ten groups to a formulation, we created an ‘others’ category in the training dataset. For each formulation, a random integer between 3 and 91 (Figure 1a), corresponding to the observed minimum and maximum numbers of KFOs per formulation, was selected. The corresponding number of KFOs was randomly sampled, assigned weights at random, and then normalized so that the sum equals 1. Each generated formulation was represented as a sparse vector over all KFOs. To avoid potential overlaps and ensure divergence from existing formulations, the average similarity to all empirical formulations was computed, and only formulations with an average similarity below 0.2 were retained. Using this procedure, 500 formulations were generated for model training.

**4. Benchmark Comparison for Generative Models**

We conducted a benchmark of three conditional generative architectures for food aroma formulation design, including conditional variational autoencoder (CVAE), conditional generative adversarial networks (GAN), and conditional denoising diffusion probabilistic models (DDPM). The primary objective is to identify a practically suitable generative model to support subsequent food formulation design and generation.

The benchmark dataset comprised 2,376 formulations (Table S3). The models based on the three architectures were trained and evaluated using an identical stratified 80:20 train-validation split generated with random seed 42. For a fair comparison, the same split, random seed, batch size, optimizer family, learning rate, scheduler, early stopping, gradient clipping threshold, conditioning information, candidate budget, and post-processing procedure were used. The three models were evaluated using checkpoints trained only on the shared training split. The final model subsequently retrained on the complete dataset was not used in the benchmark evaluation.

The CVAE used class-conditioned encoder and decoder networks, in which the one-hot category vector was concatenated with the formulation vector at the encoder input and with the latent vector at the decoder input. The model was trained by minimizing a weighted combination of reconstruction loss and Kullback-Leibler divergence. The conditional GAN consisted of a generator that received Gaussian noise together with the class condition and a discriminator that evaluated whether a formulation matched the corresponding condition. The DDPM was trained to predict additive Gaussian noise in the formulation space using a denoising network conditioned on timestep embeddings and food-category labels.

For each architecture, 1,000 candidates were generated for each of the 11 classes, resulting in 11,000 generated candidates per model. All candidates were subjected to the same sparsity-preserving post-processing procedure. Specifically, an integer k between 5 and 20 was sampled, the k largest components were retained, all remaining components were set to zero, and the resulting vector was renormalized to sum to one. We then evaluated two conditions: an unfiltered condition, in which all sparsified candidates were retained, and a filtered condition, in which candidates were retained only if the pretrained multilayer perceptron classifier assigned the generated formulation to the intended target class with probability greater than 0.95. The classifier-based filter was used only as an operational screen for category consistency and was not treated as an independent measure of generative quality.

Model performance was assessed using validation-set reference statistics. The primary metrics included the L2 distance between generated and validation class-mean vectors, the L2 distance between generated and validation class-standard-deviation vectors, the nearest-validation L2 distance, and the nearest-training L2 distance. For the filtered output dataset, both macro- and sample-weighted nearest-validation distances were reported. These metrics jointly evaluated whether the generated formulations reproduced the central tendency and variability of the empirical formulations, and whether they remained close to the observed formulation space after generation and classifier-based filtering.

Without classifier-based filtering, each model generated 11,000 candidates (Table S4). The DDPM achieved the smallest mean L2 distance (0.21), indicating the closest match to validation-set mean composition vectors. However, this advantage was limited to the class-mean level and did not extend to variability or local proximity. The DDPM showed the largest standard-deviation L2 distance (0.54), nearest-validation L2 distance (0.41), and nearest-training L2 distance (0.41). In contrast, the CVAE achieved the best standard-deviation matching (0.43), the shortest nearest-validation L2 distance (0.32), and the shortest nearest-training L2 distance (0.29). These results indicate that although the DDPM slightly better reproduced class-level mean vectors, the CVAE generated formulations that were closer to empirical formulations and better preserved class-level variability. The GAN showed intermediate nearest-neighbor performance but weaker overall distributional fidelity than the CVAE.

With classifier-based filtering, the models differed substantially in retained candidate yield and formulation fidelity (Table S4). The CVAE retained 2,182 candidates, compared with 982 candidates for the DDPM and 135 candidates for the GAN. Among retained candidates, the CVAE achieved the smallest mean L2 distance (0.22), standard-deviation L2 distance (0.45), macro nearest-validation L2 distance (0.29), weighted nearest-validation L2 distance (0.29), and nearest-training L2 distance (0.25). The DDPM retained more candidates than the GAN but showed larger distances than the CVAE across all filtered metrics, including mean L2 distance (0.30), standard-deviation L2 distance (0.56), macro nearest-validation L2 distance (0.46), and nearest-training L2 distance (0.46). The GAN retained the fewest candidates and showed weaker post-filtering performance. Overall, the CVAE provided the strongest balance between usable candidate yield and proximity to the empirical formulation space, supporting its selection for subsequent aroma formulation generation.

The benchmark revealed complementary strengths among the tested architectures. The DDPM best reproduced class-level mean vectors before filtering and achieved broad nominal post-filter coverage, suggesting that diffusion-based formulation models may become competitive after model-specific tuning. However, its poorer variance matching, larger nearest-neighbor distances, and concentration of retained candidates in one class indicated less balanced performance under the present baseline. The GAN performed poorly under this sparse, high-dimensional compositional setting, particularly after filtering. By contrast, the CVAE achieved the strongest overall balance of distributional fidelity, local plausibility, uniqueness, simplex validity, and usable high-confidence candidate yield. On this basis, the CVAE was selected as the primary architecture for subsequent hyperparameter tuning and targeted aroma formulation generation.

**5. Sensory evaluation**

In Section 1, to evaluate whether AI-generated aroma formulations were perceptually recognizable, 100 formulations were selected from model outputs across ten food categories. Within each category, ten formulations were randomly sampled using a fixed random seed (42) to ensure reproducibility (Supporting Data 2). All samples were prepared according to their generated recipes and diluted to 5% (w/w) in 1,2-propanediol. Samples were presented in identical containers coded with three-digit blind identifiers.

Testing was conducted in a well-ventilated laboratory. Before the experiment, participants were fully informed of the procedures and confirmed their voluntary participation. During evaluation, each participant was presented with a scented strip corresponding to a coded sample and instructed to select the matching food categories from a set of 11 predefined options (10 food categories plus ‘others’). For each sample, participants could select one to three categories, marking the selected categories as 1 and the unselected categories as 0.

In Section 2, to assess whether meat-like aromas could be reconstructed using exclusively plant-derived KFOs (e.g., those present in coffee, cocoa, tea, fruits, grain products, legumes, nuts, oilseeds, spices, and vegetables), three formulations were randomly selected from 730 generated “meat” candidates composed only of plant-origin KFOs and designated as Samples 1-3. Sample 3 was duplicated to assess panel consistency. A literature-derived reconstituted meat aroma formulation was included as a meat aroma reference for comparison. All formulations were prepared according to their recipes (Table S6) and diluted to 5% (w/w) in 1,2-propanediol. All compounds were sourced from Shanghai Meixin Chemical Technology Co., Ltd. A blank sample (air) and a matrix sample (1,2-propanediol) were also included, resulting in a total of seven samples for evaluation. Participants first classified each aroma sample as either “with meat aroma” or “without meat aroma”. Subsequently, they rated each sample across eight dimensions of the aroma wheel, following the design of Wang et al.^1^, including umami, garlic-like, sauce-like, green, salty, sweet, meaty, and fatty, using a 1-9 scale.

For hedonic evaluation, to minimize the influence of movement on flavor perception, aromas were delivered to participants’ faces at a flow rate of 5 L/min using a gas pump positioned 5-7 cm from the nose. The experimenter first stabilized aroma delivery with the pump, then provided a standardized plant-based ham sausage sample, instructing participants to chew and swallow. Immediately after swallowing, participants rated hedonic response using the SAM scale^2^. To investigate the contribution of orthonasal olfaction to hedonic perception, a control condition was included in which participants wore nasal clips to block orthonasal airflow while performing the same tasting and rating procedure. To prevent cumulative effects of taste and smell, a 5-minute interval was set between trials, during which participants were given small amounts of warm water and briefly removed from the testing area to clear the oral and nasal passages. Plant-based ham sausages were purchased from Shenzhen Whole Perfect Foods Co., Ltd., contained no added aroma, and were uniformly cut into 1.5 cm pieces.

Panelists were not formally trained prior to the sensory evaluation. This choice was motivated by practical considerations. Unlike well-defined aromas such as “apple” or “vanillin”, the target stimuli in this study correspond to broad and chemically heterogeneous food categories (e.g., fruits, vegetables, beverages, and meat), for which no standardized reference compounds or canonical training samples exist. In addition, the aroma identification (e.g., meat-like versus non-meat-like) focuses on category-level recognition and does not require fine-grained, calibrated descriptor scaling or expert cue learning. These tasks rely on holistic perceptual assessment rather than analytical discrimination. Therefore, we used experienced (having participated in at least 3 sensory evaluations previously in our lab) but untrained panelists to provide a conservative test of whether the learned KFO organizations translate into perceptually recognizable aroma identities under natural smelling conditions.

To further evaluate the reliability of the sensory data, inter-rater agreement among panelists was assessed separately for meat aroma recognition, aroma profile evaluation, and hedonic evaluation. For the binary meat aroma recognition task, agreement was quantified using Fleiss’ kappa based on the Yes/No classifications. The overall Fleiss’ kappa was 0.65, with a mean observed agreement of 0.83, indicating substantial agreement among panelists. For the aroma profile ratings, inter-rater reliability was estimated using intraclass correlation coefficients under an absolute-agreement framework. The single-rater ICC ranged from 0.56 to 0.82 across sensory attributes, with a mean value of 0.68, indicating moderate to good agreement among individual panelists. When ratings were averaged across the 30 panelists, the average-measure ICC ranged from 0.97 to 0.99, with a mean value of 0.98, demonstrating excellent reliability of the averaged aroma profile scores. For pleasantness evaluation, the single-rater ICC was 0.79, indicating good agreement among panelists, while the average-measure ICC was 0.99, supporting the high reliability of the mean hedonic scores used for subsequent analysis.

**6. Electroencephalography assessment**

Participants were seated comfortably in ergonomically designed chairs. Odors of sample 3, the literature-derived reconstituted meat aroma reference, and air were delivered using a gas pump at a flow rate of 5 L/min, positioned 5- 7 cm in front of the participant’s face (Figure S5a). EEG recordings consisted of a 30-second resting period followed by a 60-second odor-exposure period (Figure S5b). Participants were instructed to press the green button on the SAGA synchronization module immediately upon perceiving the aroma, marking the onset of the exposure period.

EEG data were acquired using a TMSI SAGA EEG device (Twente Medical Systems International BV, the Netherlands) with a 64-channel cap arranged according to the international 10-20 system (Figure S5c) and sampled at 500 Hz. Electrode impedance was maintained below five kΩ.

Preprocessing was performed in MATLAB using the EEGLAB toolbox ^3^. Signals were band-pass filtered between 1 and 70 Hz, and a 50 Hz notch filter was applied to remove power-line interference. Independent component analysis was used to remove artifacts, including electrooculographic and electromyographic activity. Channels M1 and M2 were excluded, and an average reference was applied. Based on the recorded event times, the subsequent 30 s of data were extracted as aroma exposure segments, which were further divided into 2 s epochs with 50% overlap ^4^.

All subsequent analyses were performed using the MNE-Python software package ^5^. To ensure accurate visualization and source modeling, the standard fsaverage brain template was used, providing a normalized cortical surface and source space for group-level analysis ^6^.

For source localization, forward models and noise covariance matrices were computed separately for each experimental condition. Forward models were generated using a boundary element model based on the fsaverage template, with the source space defined on the cortical surface of fsaverage, containing 10,242 vertices per hemisphere at ico-5 resolution. Forward models combined EEG sensor locations, a boundary element model, and the source space, with a minimum source spacing of 5 mm to avoid closely spaced sources. Noise covariance matrices were estimated primarily from the full set of epochs, with regularization parameters optimized automatically. Source localization was performed using dynamic statistical parametric mapping.

For each condition, EEG epoch data were loaded from preprocessed files and aligned to the standard 10-20 electrode layout. The inverse operator was configured with loose orientation constraints (loose=0.2) and depth weighting (depth=0.8) to account for deeper cortical sources. A signal-to-noise ratio (SNR) of 3.0 was assumed to calculate the regularization parameter (λ²=1/SNR²). Source time courses (STCs) were computed for each epoch and subsequently averaged across epochs within each condition to produce mean STCs for statistical analysis.

To examine differences in brain activity across conditions, the mean STCs were compared using correlation analyses. For each vertex in the source space, time series from two conditions were compared using Pearson or Spearman correlation, depending on data normality, assessed with the Shapiro-Wilk test. Given the exploratory nature of this analysis, vertex-wise significance was used to define candidate regions, and results are interpreted at the level of regional patterns rather than single-vertex inference. Vertices with p < 0.05 were identified as significant. Significant vertices were mapped to anatomical labels using the aparc parcellation of the fsaverage template, and for each label, the mean correlation coefficient and number of significant vertices were calculated.

For visualization, source-space similarity maps were created in MNE-Python using correlation coefficients as values. Maps were rendered on the fsaverage cortical surface with a symmetrical color scale from -1 to 1. Four 3D views (left lateral, right lateral, dorsal, and ventral) were generated using the PyVista backend^7^.

**Figure S1. Structural characterization of KFO compositions across food categories.** Heatmaps summarize the distribution of 18 chemical groups across 17 food categories, quantified by (a) mean occurrence frequency per KFO formulation, (b) mean relative concentration within each category, and (c) log₁₀-transformed odor activity values (OAVs). Structural classes are ordered from top to bottom by their overall abundance across categories, facilitating comparisons of dominant and rare compound classes. White cells indicate the absence of the corresponding chemical groups in a given category.

**Figure S2. Chemical class composition across frequency-defined KFO groups.** Nested donut chart showing the chemical class composition of KFOs stratified by frequency across formulations. KFOs were classified into generalists (present in >25% of samples), intermediaries (5-25%), and individualists (<5%). The outer ring indicates the total number of KFOs in each frequency class, whereas the inner segments depict the relative contributions of different chemical classes. Generalists are confined to a small number of chemically simple classes, predominantly alcohols, aldehydes, and esters. Intermediaries span a broader range of structural classes, while individualists encompass all chemical classes.

**Figure S3.** **Validation and sensitivity analysis of the compositional similarity metric.** (a) Spearman correlation heatmap comparing category-level homogeneity scores obtained using the composite cosine-Manhattan metric and six alternative similarity metrics, including cosine, normalized Manhattan, Bray-Curtis, Jaccard, Canberra, and Euclidean similarity. Asterisks indicate statistical significance of the rank correlations (*, p<0.05; **, p<0.01; ***, p<0.001). (b) Equivalence between Earth Mover’s Distance (EMD) and normalized L1 distance under a discrete-uniform ground metric. Because no chemically or perceptually defined KFO-level distance matrix was available, EMD reduces to the same information captured by the normalized Manhattan component of the composite score. (c) Mean within-category similarity across food categories as a function of the cosine weight (w) in the combined metric, where w=0.50 represents the baseline equal-weight setting. (d) Kendall’s τ comparing category-level homogeneity rankings across different cosine weights with the baseline ranking at w=0.50. The stability of rankings across a broad weight range supports the robustness of the equal-weight composite cosine-Manhattan similarity metric.

**Figure S4.** Training loss of the conditional variational autoencoder under the optimal hyperparameter configuration.

**Figure S5. Performance evaluation of the multilayer perceptron models under stratified individual-profile-level validation.** (a) One-vs-rest receiver operating characteristic (ROC) curves for the multilayer perceptron model. (b) Precision-recall curves for the multilayer perceptron model. (c) Confusion matrix of the multilayer perceptron classifier for category-level prediction. Food categories (Classes 0-10) correspond to: alcoholic beverages, fats and oils, coffee/cocoa/tea, fish and seafood, fruit products, grain products, legumes/nuts/oilseeds/spices, meat products, seasonings and condiments, vegetable products, and others.

**Figure S6. Repeatability assessment of the sensory evaluation (n=30).** (a) Sensory panel judgments of the meat aroma of sample 3 and its duplicate. (b) Pleasantness scores for sample 3 and its duplicate, showing no significant differences. (c) Sensory intensity scores for sample 3 and its duplicate also showed no significant differences.

Note: In panels (b) and (c), the blue and orange scatter points denote individual ratings assigned by participants to each sample across different sensory dimensions. Grey box plots summarize the distributions for each group, displaying the first and third quartiles, the median, and the range of non-outlying values. Dark red points indicate the group means, and black curves represent the corresponding probability density distributions.

**Figure S7.** **Electroencephalography response-pattern similarity to the literature-derived meat aroma reference.** Violin plots show the distribution of Spearman correlation coefficients calculated across cortical regions between the literature-derived reconstituted meat aroma reference and the AI-generated Sample 3, or the air condition.

Note: Each blue dot represents one cortical region. Box plots indicate the median and interquartile range, and orange dots denote the mean correlation coefficient for each comparison.

**Figure S8. Experimental setup for electroencephalography (EEG).** (a) Schematic of the aroma delivery system used during plant-based sausage tasting and EEG recordings. (b) Workflow of the EEG experiment. (c) Illustration of the 10-20 EEG electrode distribution.

**Table S1. Number and proportion of KFOs classified by chemical groups.**

| **Chemical groups** | **Freqs** | **Proportions** |
| --- | --- | --- |
| Esters | 179 | 20.0% |
| Alcohols | 121 | 13.5% |
| Carbonyls, aldehydes | 99 | 11.0% |
| Carbonyls, ketones | 98 | 10.9% |
| Sulfur compounds | 91 | 10.2% |
| Hydrocarbons | 77 | 8.6% |
| Bases | 58 | 6.5% |
| Furans | 39 | 4.4% |
| Acids | 34 | 3.8% |
| Phenols | 31 | 3.5% |
| (Ep)oxides, pyrans, coumarins | 27 | 3.0% |
| Lactones | 23 | 2.6% |
| Nitriles and amides | 9 | 1.0% |
| Ethers | 4 | 0.4% |
| Acetals | 2 | 0.2% |
| Halogens | 2 | 0.2% |
| Imidazoles | 1 | 0.1% |
| Oxazol(in)es | 1 | 0.1% |

**Table S2. Number of KFOs per aroma profiles across food categories.**

| **Food categories** | **Mean** | **Max** | **Min** |
| --- | --- | --- | --- |
| Alcoholic beverages | 13.38 | 57 | 3 |
| Coffee, cocoa and tea | 16.49 | 91 | 4 |
| Fruit products | 11.45 | 46 | 3 |
| Vegetable products | 13.71 | 37 | 4 |
| Fats and oils | 14.02 | 37 | 3 |
| Seasoning and condiments | 17.05 | 48 | 3 |
| Juices and nectars | 12.14 | 33 | 3 |
| Fish and seafood | 10.63 | 27 | 3 |
| Meat products | 14.88 | 33 | 3 |
| Composite dishes | 15.91 | 43 | 3 |
| Grain products | 11.71 | 45 | 3 |
| Legumes, nuts, oilseeds and spices | 17.02 | 47 | 4 |
| Food imitates and supplements | 13.12 | 34 | 3 |
| Milk and dairy products | 11.29 | 25 | 3 |
| Sugar and confectionery products | 13.27 | 24 | 3 |
| Major isolated ingredients and additives | 13.53 | 22 | 7 |
| Water-based beverages | 9.36 | 13 | 5 |

**Table S3. Number of formulations for ten balanced, data-rich food categories.**

| **Food categories** | **Counts** |
| --- | --- |
| Alcoholic beverages | 546 |
| Fats and oils | 132 |
| Coffee, cocoa and tea | 236 |
| Fish and seafood | 125 |
| Fruit products | 200 |
| Grain products | 117 |
| Legumes, nuts, oilseeds and spices | 99 |
| Meat products | 125 |
| Seasoning and condiments | 130 |
| Vegetables products | 166 |
| Others | 500 |

**Table S4. Benchmark performance after shared sparsification, before and after Multilayer perceptron classifier filtering at a probability threshold of > 0.95.**

| **Datasets** | **Metrics** | **CVAE** | **GAN** | **DDPM** |
| --- | --- | --- | --- | --- |
| Unfiltered | Generated candidates | 11000 | 11000 | 11000 |
|  | Mean L2 distance | 0.22 | 0.27 | 0.21 |
|  | Standard-deviation L2 distance | 0.43 | 0.48 | 0.54 |
|  | Nearest-validation L2 distance | 0.32 | 0.37 | 0.41 |
|  | Nearest-training L2 distance | 0.29 | 0.36 | 0.41 |
| Filtered | Retained candidates | 2182 | 135 | 982 |
|  | Mean L2 distance | 0.22 | 0.34 | 0.30 |
|  | Standard-deviation L2 distance | 0.45 | 0.53 | 0.56 |
|  | Macro nearest-validation L2 distance | 0.29 | 0.38 | 0.46 |
|  | Weighted nearest-validation L2 distance | 0.29 | 0.39 | 0.32 |
|  | Nearest-training L2 distance | 0.25 | 0.37 | 0.46 |

**Table S5. Hyperparameter search space for optimizing the conditional variational autoencoder.**

| **Hyperparameters** | **Search ranges** | **Bests** |
| --- | --- | --- |
| latent_dim | [16, 32, 64, 128] | 64 |
| learning_rate | [1e-5, 5e-5, 1e-4, 5e-4] | 0.0005 |
| patience | [20, 30, 50] | 50 |
| recon_weight | [0.5, 1.0, 2.0] | 0.5 |
| kl_weight | [0.1, 0.5, 1.0] | 0.1 |
| sim_weight | [0.5, 1.0, 2.0] | 0.5 |
| beta_min | [0.05, 0.1, 0.2] | 0.05 |
| beta_max | [0.5, 1.0, 2.0] | 0.5 |
| beta_cycle | [50, 100, 200] | 200 |
| encoder_hidden | [512, 256]; [256, 128]; [512, 256, 128] | [512, 256, 128] |
| decoder_hidden | [256, 512]; [128, 256]; [128, 256, 512] | [256, 512] |

**Table S6. Summary of AI-generated aroma formulations by food category (classifier probability > 0.95), and their mean maximum similarity to existing aroma formulations.**

| **Categories** | **Counts** | **Mean maximum similarity** |
| --- | --- | --- |
| Alcoholic beverages | 997 | 0.73 |
| Fats and oils | 904 | 0.60 |
| Coffee, cocoa, tea | 823 | 0.55 |
| Fish products | 951 | 0.73 |
| Fruit products | 803 | 0.60 |
| Grain products | 827 | 0.61 |
| Legumes, nuts, oilseeds and spices | 712 | 0.53 |
| Meat products | 730 | 0.68 |
| Seasoning and condiments | 842 | 0.72 |
| Vegetable products | 825 | 0.52 |
| Others | 970 | 0.11 |

**Table S7. AI-generated aroma formulations (samples 1-3) and the literature-derived reconstituted meat aroma reference used for sensory evaluation.**

| **Samples** | **PubChem IDs** | **Compound names** | **Proportions** |
| --- | --- | --- | --- |
| Sample 1 | 179 | 3-hydroxy-2-butanone | 0.1436 |
|  | 264 | butanoic acid | 0.0159 |
|  | 454 | 1-octanal | 0.0039 |
|  | 7144 | creosol | 0.0243 |
|  | 7165 | ethyl benzoate | 0.1281 |
|  | 7342 | ethyl isobutyrate | 0.0077 |
|  | 7762 | ethyl butanoate | 0.0600 |
|  | 7797 | ethyl heptanoate | 0.0269 |
|  | 7799 | ethyl octanoate | 0.0804 |
|  | 7824 | methyl hexanoate | 0.0170 |
|  | 8048 | ethyl decanoate | 0.0780 |
|  | 12398 | heptadecane | 0.0137 |
|  | 25611 | 2-methyl-3-heptanone | 0.0058 |
|  | 31249 | diethyl succinate | 0.0060 |
|  | 31253 | beta-Myrcene | 0.0072 |
|  | 31265 | ethyl hexanoate | 0.1674 |
|  | 88290 | 4-methyl-4-mercaptopentan-2-one | 0.0042 |
|  | 440917 | D-limonene | 0.2101 |
| Sample 2 | 798 | indole | 0.0081 |
|  | 7144 | creosol | 0.0163 |
|  | 7165 | ethyl benzoate | 0.1437 |
|  | 7762 | ethyl butanoate | 0.0439 |
|  | 7797 | ethyl heptanoate | 0.0316 |
|  | 7799 | ethyl octanoate | 0.0701 |
|  | 7824 | methyl hexanoate | 0.0075 |
|  | 8048 | ethyl decanoate | 0.0606 |
|  | 12398 | heptadecane | 0.0237 |
|  | 26808 | 2,3,5-trimethylpyrazine | 0.0038 |
|  | 31249 | diethyl succinate | 0.0031 |
|  | 31253 | beta-Myrcene | 0.0122 |
|  | 31265 | ethyl hexanoate | 0.1233 |
|  | 88290 | 4-methyl-4-mercaptopentan-2-one | 0.0042 |
|  | 440917 | D-limonene | 0.4444 |
|  | 853433 | isoeugenol | 0.0035 |
| Sample 3 | 179 | 3-hydroxy-2-butanone | 0.0324 |
|  | 264 | butanoic acid | 0.0326 |
|  | 454 | 1-octanal | 0.0552 |
|  | 460 | guaiacol | 0.0211 |
|  | 998 | phenylacetaldehyde | 0.1266 |
|  | 3314 | eugenol | 0.0255 |
|  | 6184 | 1-hexanal | 0.0919 |
|  | 6549 | linalool | 0.0247 |
|  | 8063 | pentanal | 0.0509 |
|  | 8103 | 1-hexanol | 0.0438 |
|  | 8130 | 1-heptanal | 0.0457 |
|  | 11552 | 3-methyl butanal | 0.0935 |
|  | 18827 | 1-octen-3-ol | 0.0836 |
|  | 19309 | furaneol | 0.0205 |
|  | 19310 | dimethyl trisulfide | 0.0216 |
|  | 31265 | ethyl hexanoate | 0.0322 |
|  | 31289 | nonanal | 0.1335 |
|  | 637563 | anethole | 0.0216 |
|  | 5283345 | (E)-2-decenal | 0.0428 |
| Reference | 179 | 3-hydroxy-2-butanone | 0.0749 |
|  | 323 | coumarin | 0.0119 |
|  | 454 | 1-octanal | 0.0032 |
|  | 1136 | sulfurol | 0.2165 |
|  | 2758 | 1,8-cineole | 0.0844 |
|  | 2879 | p-cresol | 0.0003 |
|  | 3314 | eugenol | 0.0868 |
|  | 6184 | 1-hexanal | 0.0045 |
|  | 6549 | linalool | 0.0181 |
|  | 6654 | alpha-pinene | 0.0053 |
|  | 6920 | 2-acetylthiophene | 0.0045 |
|  | 8063 | pentanal | 0.0011 |
|  | 8103 | 1-hexanol | 0.0614 |
|  | 8129 | 1-heptanol | 0.0027 |
|  | 8130 | 1-heptanal | 0.0014 |
|  | 8815 | estragole | 0.0023 |
|  | 18635 | methional | 0.0013 |
|  | 19309 | furaneol | 0.0020 |
|  | 19310 | dimethyl trisulfide | 0.1732 |
|  | 21059 | ethyl maltol | 0.0084 |
|  | 31244 | 4-methoxybenzaldehyde | 0.0110 |
|  | 31289 | nonanal | 0.0470 |
|  | 34286 | 2-methyl-3-furanthiol | 0.0024 |
|  | 61346 | 1-octen-3-one | 0.0023 |
|  | 83101 | 2,6-diethylpyrazine | 0.0020 |
|  | 520108 | 2-acetylthiazole | 0.0025 |
|  | 637563 | anethole | 0.1420 |
|  | 4564493 | 5-methyl-4-hydroxy-3(2H)-furanone | 0.0035 |
|  | 5282110 | cinnamyl acetate | 0.0088 |
|  | 5283345 | (E)-2-decenal | 0.0060 |
|  | 5283349 | 2,4-decadienal | 0.0025 |
|  | 5283356 | (E)-2-undecenal | 0.0058 |

**Table S8. Comparison of machine learning algorithms to predict aroma categories based on the proportion of KFOs as input features.**

| **Machine learning algorithms** | **F1 scores** ± **standard deviation** |
| --- | --- |
| Decision trees | 0.78 ± 0.02 |
| Linear discriminant analysis | 0.77 ± 0.01 |
| Logistic regression | 0.86 ± 0.01 |
| Multilayer perceptron | 0.87 ± 0.02 |
| Support vector machines | 0.86 ± 0.02 |

**References**

1	Wang, Z. *et al.* Decoding the flavor of traditional Chinese stir-fried pork cuisine: Development of a sensory wheel and exploration of the relationship between sensory attributes and aroma compounds. *Food Res Int* **214**, 116650 (2025). <https://doi.org/10.1016/j.foodres.2025.116650>

2	Bradley, M. M. & Lang, P. J. Measuring emotion: The self-assessment manikin and the semantic differential. *Journal of Behavior Therapy and Experimental Psychiatry* **25**, 49-59 (1994). <https://doi.org/https://doi.org/10.1016/0005-7916(94)90063-9>

3	Delorme, A. & Makeig, S. EEGLAB: an open source toolbox for analysis of single-trial EEG dynamics including independent component analysis. *Journal of neuroscience methods* **134**, 9-21 (2004).

4	Gil Ávila, C. *et al.* DISCOVER-EEG: an open, fully automated EEG pipeline for biomarker discovery in clinical neuroscience. *Scientific Data* **10**, 613 (2023).

5	Gramfort, A. *et al.* MEG and EEG data analysis with MNE-Python. *Frontiers in Neuroinformatics* **7**, 267 (2013).

6	Fischl, B. FreeSurfer. *NeuroImage* **62**, 774-781 (2012). <https://doi.org/https://doi.org/10.1016/j.neuroimage.2012.01.021>

7	Sullivan, C. & Kaszynski, A. PyVista: 3D plotting and mesh analysis through a streamlined interface for the Visualization Toolkit (VTK). *Journal of Open Source Software* **4** (2019). <https://doi.org/10.21105/joss.01450>
